# Mechanisms behind *idr1–1* mutation conferring osmotic-stress tolerance to rice seedlings as revealed by stage-based transcriptomes

**DOI:** 10.3389/fpls.2026.1815995

**Published:** 2026-05-13

**Authors:** Tiange Hu, Yi Zhou, Yan Liu, Yifei Sun, Rongdi Yang, Fei Chen, Xianwen Zhang, Waseem Hussain, Mohamed Abdel-Gawad Emam, Zhaoyu Zhai, Shaoxia Zhou, Honggui La

**Affiliations:** 1 College of Life Sciences, Nanjing Agricultural University, Nanjing, China; 2 Institute of Virology and Biotechnology, Zhejiang Academy of Agricultural Sciences, Hangzhou, China; 3 International Rice Research Institute (IRRI), Los Baños, Laguna, Philippines; 4 Faculty of Agriculture, Suez Canal University, Ismailia, Egypt; 5 College of Smart Agriculture (College of Artificial Intelligence), Nanjing Agricultural University, Nanjing, China; 6 College of Plant Protection, Nanjing Agricultural University, Nanjing, China

**Keywords:** chloroplast, differentially expressed genes (DEGs), increased drought resistance 1 (*IDR1*), osmotic-stress tolerance, redox homeostasis, stage-dependent transcriptomic analysis

## Abstract

Osmotic stress, which is mainly caused by water deficiency, is one of the major environmental factors limiting rice productivity. Osmotic stress influences many aspects of plant growth and development, especially flowering. The α subunit of the heterotrimeric G protein complex, IDR1 (also known as RGA1), has been reported to be involved in multiple abiotic-stress responses, while its role in coping with osmotic stress remains unclear. Here, we performed stress stage-based transcriptomic analyses of rice leaves from *idr1–1* mutant and wild-type IAPAR9 seedlings that underwent early or middle stage of osmotic stress induced by 20% PEG solution, in order to ascertain the differences in transcriptomes between *idr1–1* mutant and IAPAR9 seedlings following early- or middle-stage osmotic stress. Our results showed that 2881 upregulated and 2191 downregulated differentially expressed genes (DEGs) were identified in *idr1–1* mutant seedlings relative to wild-type IAPAR9 seedlings under early-stage osmotic stress. Similarly, 2824 upregulated and 2153 downregulated DEGs were also detected in *idr1–1* mutant seedlings relative to IAPAR9 seedlings under middle-stage osmotic stress. Overlap analyses revealed that 44 and 325 DEGs were found in *idr1–1* mutant seedlings under early and middle stages of osmotic stress, respectively, which were co-regulated by both *idr1–1* mutation and osmotic stress. Gene Ontology (GO) analyses of these DEGs demonstrated that in *idr1–1* mutant seedlings, GO terms were mainly associated with quick responses to stress (including responses to phytohormones, scavenging of ROS and stomatal movement) following early-stage osmotic stress, while those were associated with operation of photosynthetic systems (including assembly and repair of photosystem complexes, chlorophyll catabolism, and thylakoid) following middle-stage osmotic stress. Interaction assays indicated that IDR1 was able to interact with 5 proteins, OsFLU1, OsHHO3, OsRLIN1, NADPH HC and OsS40-14, with their gene expression being also regulated by *idr1–1* mutation. Altogether, our results suggest that *idr1–1* mutation contributes to enhanced tolerance to osmotic stress by altering responsiveness to different physiological processes, like responses to water deficit, salt and heat stresses, phytohormone signaling, ROS scavenging, biosynthesis of secondary metabolites, and maintenance and repair of photosynthetic systems, which may play essential roles in enabling *idr1–1* mutant seedlings to survive persistent osmotic stress.

## Introduction

Drought stress is one of the most severe environmental constraints limiting rice productivity worldwide, particularly in upland and rainfed ecosystems (Liu et al., 2022; Sato et al., 2024). With the increase in climate variability, the frequency and duration of drought events pose a major challenge to sustainable rice production (He et al., 2014). Recent years, other climate-related factors such as fluctuations in temperature and rainfall also significantly affect rice yield stability in particular areas (Kumar et al., 2025). Breeding of rice varieties with enhanced drought tolerance has therefore become an urgent objective for breeding programs (Zhu et al., 2024). However, drought tolerance in rice is a complex quantitative trait that requires coordinated regulation of physiological, molecular, and metabolic processes during stress (Khan et al., 2021). Understanding the molecular determinants underlying drought adaptation remains a central focus in plant stress biology (Song et al., 2026).

At the physiological level, drought rapidly alters plant water status, stomatal conductance, and photosynthetic efficiency, thereby leading to metabolic reprogramming and growth inhibition (Tavu and Redillas, 2025). At the molecular level, drought triggers extensive transcriptional reprogramming that involves hormone signaling, reactive oxygen species (ROS) homeostasis, and cellular protection pathways (Zheng et al., 2026). Importantly, plant responses to drought stress are highly dynamic and often stage-dependent. Early stress perception typically activates signaling cascades associated with abscisic acid (ABA) accumulation, ROS bursts, calcium influx, and stomatal closure, which subsequently induce protective gene expression (Kesawat et al., 2023; Zheng et al., 2026). As drought persists, plants gradually shift from rapid stress responses to long-term acclimation processes involving photosynthetic regulation, osmotic adjustment, detoxification, and aging (Cao et al., 2020; Kim et al., 2024; Jiang et al., 2025). Despite extensive efforts, the molecular mechanisms whereby plants shift between stress stages to maintain homeostasis during prolonged stress remain largely elusive.

So far, many drought stress-induced factors have been identified in rice, including those involved in ABA signaling, transcriptional regulation, and osmotic adjustment (Wang et al., 2001; Fan et al., 2008; Zhang et al., 2008; Nilson and Assmann, 2010; Chakravorty et al., 2011). However, most of these regulators acted mainly during the early stage of stress responses, and only a few were known to influence transcriptional or metabolic regulation under prolonged drought stress conditions (Chen et al., 2020; Gong et al., 2025). Therefore, stage-based investigations are needed to understand dynamic changes in gene expression during stress progression and to identify key factors that sustain long-term drought tolerance (Huo et al., 2024; Iqbal et al., 2025). Unraveling transcriptomic alterations during drought-stress progression can help identify the key regulators functioning in stage-dependent drought tolerance.

Chloroplasts play pivotal roles in both stress sensing and tolerance (Schwenkert et al., 2022). In addition to their roles in photosynthesis, chloroplasts are major sites for ROS generation and redox signaling under stress conditions (Zait et al., 2021; Li and Kim, 2022). During drought stress, stomatal closure limits CO_2_ uptake, thereby causing over-reduction of photosynthetic electron transport chain and increased ROS production (Martarello et al., 2024). These ROS act not only as damaging agents, but also as signaling intermediates that activate downstream defenses and acclimation pathways. Maintaining chloroplast integrity and redox homeostasis is therefore critical for drought tolerance (Wang et al., 2023). These processes are also likely to be important for retaining stress tolerance under prolonged water-deficit conditions.

Our previous investigations identified *IDR1* via a map-based cloning approach (Zu et al., 2021). *IDR1* encodes the α subunit of a heterotrimeric G-protein complex, also known as *RGA1*, which functions as a conserved molecular switch mediating signal transduction between external stimuli and intracellular effectors (Zu et al., 2021). The *idr1–1* mutant carries an 1-bp deletion in the *IDR1* exon, thereby creating a truncated protein (Zu et al., 2021). In rice, *IDR1/RGA1* had been shown to regulate multiple developmental and stress-related processes, including seed sizes, panicle development, and responses to abiotic stresses such as drought and salinity (Zu et al., 2021). The rice *rga1* (*d1*) mutant plants exhibited enhanced drought tolerance and reduced photosynthetic inhibition under stress conditions (Ashikari et al., 1999; Ferrero-Serrano and Assmann, 2016). Physiological studies indicated that the absence of *Arabidopsis GPA1*, an ortholog of rice *RGA1*, interrupted ABA signaling between ABA reception and ROS production, thereby impairing Ca^2+^-channel activation and ROS accumulation in guard cells (Zhang et al., 2011). Our previous work showed that the *idr1–1* mutant plants displayed enhanced drought tolerance under both field and controlled conditions; *idr1–1* exhibited altered ROS-related responses under drought stress, which provided support for a role of IDR1 in redox-related stress regulation (Zu et al., 2021). Together, these findings indicate that IDR1 acts as a negative regulator of drought tolerance, possibly through modulation of ROS homeostasis and phytohormone signaling.

Although *IDR1*/*RGA1* has been implicated in drought tolerance, its role in osmotic-stress tolerance and the underlying molecular mechanisms remain largely unknown. Therefore, we used PEG-induced osmotic stress to cause cellular dehydration in order to ascertain whether *idr1–1* mutation confers enhanced tolerance to the dehydration in rice seedlings. In this study, we examined the tolerance of *idr1–1* mutant seedlings to osmotic stress, and found that they exhibited enhanced tolerance to this type of stress. To further understand the molecular basis of the tolerance enhancement at the transcriptomic level, we used stage-based transcriptomic analysis to examine responses of the *idr1–1* mutant and wild-type IAPAR9 seedlings to osmotic stress induced by 20% PEG solution. By comparing differences in differentially expressed genes (DEGs) between *idr1–1* mutant and wild-type IAPAR9 seedlings, as well as shifts in DEG categories from early-stage stress to middle-stage stress, we identified a large number of DEGs in the *idr1–1* mutant seedlings undergoing early-stage or middle-stage osmotic stress compared with the wild-type IAPAR9 seedlings without osmotic stress. Furthermore, 44 DEGs and 325 DEGs co-regulated by the *idr1–1* mutation and osmotic stress were identified from early-stage and middle-stage stress treatments, respectively. Under prolonged osmotic stress, *idr1–1* mutation appeared to facilitate alterations in redox status and photosynthetic function, thereby reducing the effects of osmotic stress. In addition, IDR1 was also found to interact with OsFLU1, OsHHO3, OsRLIN1, NADPH HC and OsS40-14, which were all co-regulated by the *idr1–1* mutation and osmotic stress. Thus, these findings lay the foundation for further understanding the molecular mechanisms behind enhanced tolerance to osmotic stress observed in the *idr1–1* mutant seedlings.

## Materials and methods

### Plant materials and stress treatments

The upland rice cultivar IAPAR9 (*Oryza sativa* L. ssp. *japonica*) and the *idr1–1* mutant, which was derived from ^60^Co γ-ray radiation-mutagenized IAPAR9 seeds, were used in this study (Zu et al., 2021). The *idr1–1* mutant carried a 1-bp deletion in the fifth exon of Os05g0333200 (*IDR1*/*D1*/*RGA1*), which caused a frameshift mutation and created a premature translation termination codon in the sixth exon (Zu et al., 2021).

For osmotic-stress treatments, seeds were surface-sterilized, soaked, and germinated at 28 °C, then sown in quartz sands and irrigated with full-strength Hoagland nutrient solution. The containers for seedlings were 15 cm in height and 12 cm in diameter, and each housed 20 wild-type and 20 *idr1–1* mutant seedlings that were spaced evenly apart. The containers were placed in a growth chamber, under conditions of 16-h light/8-h dark, 28 °C, 300 μmol·m^-^²·s^-^¹ light intensity, and 70% relative humidity, for approximately 14 days (3**–**4-leaf stage). Subsequently, healthy seedlings were chosen and cleaned, and then transferred to Hoagland solution supplemented with 20% (w/v) polyethylene glycol 6000 (PEG6000) or to Hoagland solution alone, which served as untreated controls. The solutions were renewed daily to ensure sufficient nutrient supply. Phenotypic changes, including leaf rolling, yellowing, and wilting, were recorded at 0, 12, 24, 48, and 72 h after PEG treatment. For measurement of relative water contents of leaves, leaf samples were collected at the same time points and their fresh weights (FW) were determined immediately. Samples were then immersed in distilled water for 24 h at room temperature for the purpose of obtaining turgid weights (TW), followed by being dried at 80 °C to determine dry weights (DW). Relative water content was calculated by (FW−DW)/(TW−DW) × 100%. To calculate survival rates following recovery, seedlings were returned to regular Hoagland solution after 72-h PEG treatments to allow them to recover for 3 days prior to calculations of survival rates.

### Preparation of leaf samples and mRNA sequencing

For genome-wide mRNA sequencing, leaves were collected separately from IAPAR9 and *idr1–1* seedlings each undergoing 24-h or 48-h 20% PEG treatments; 24-h and 48-h 20% PEG treatments were designated as early (E) and middle (M) stages of osmotic stress, respectively. Simultaneously, leaves from the wild-type IAPAR9 and *idr1–1* mutant seedlings growing under the control conditions (i.e., in regular Hoagland solution) for 24 or 48 hours were also harvested in parallel. The leaf-derived mRNA samples isolated from three biological replicates of each genotype in control or treatment groups were used for library preparation and Illumina sequencing by Kangce (China).

### Data processing and bioinformatic analyses

Raw reads of mRNA-seq data were trimmed with Trim_galore (v0.6.10) to remove adapters and low-quality sequences. Clean reads were aligned to the Nipponbare reference genome (MSU Rice Genome Annotation Project, Release 7) using HISAT2 (v2.2.1) with default parameters, and read counts were summarized with FeatureCounts (v2.0.6). For quality assessment of different samples, raw read counts were first normalized as counts per million (CPM). Low-abundance genes were filtered out, and only those genes meeting the cutoff of CPM ≥ 1 were retained in three samples. The resulting CPM matrices were then transformed using log_10_ (CPM + 1) to improve data distribution. Subsequently, the resulting expression matrices were used to generate principal component analysis (PCA) and heatmaps of sample-to-sample correlation. Differential expression analyses were conducted with the DESeq2 R package (v1.42.1), where the input data for DESeq2 were the raw expression matrices obtained from FeatureCounts without any prior normalization. Differentially expressed genes (DEGs) were identified based on the criteria of false discovery rate (FDR) < 0.05 and |Fold Change| ≥ 1.5. Volcano plots were generated to visualize DEGs for each comparison group, and overlaps among DEG data sets were displayed in the form of Venn diagrams (up- and down-regulated genes were analyzed separately).

Gene Ontology (GO) enrichment analyses were conducted using the Rice Gene Index (RGI) website. Enrichment significance was determined by Fisher’s exact test, and adjusted *P* values were corrected by the Benjamini-Hochberg method. GO terms with adjusted *P* < 0.05 were considered significantly enriched.

### Quantitative RT−PCR analyses

Total RNA was extracted from rice tissues with TRIzol™ (R411-01, Vazyme, China) according to the manufacturer’s protocol. Residual genomic DNA was eliminated using the gDNA Remover component of the Evo M-MLV RT Kit (AG11728, Accurate Biology, China). First-strand cDNA was synthesized with oligo(dT) primers according to the supplier’s instructions. Quantitative real-time PCRs (qRT-PCRs) were carried out with the SYBR^®^ Green Pro Taq HS Premixed qPCR Kit (AG11701, Accurate Biology China) on an ABI 7500 Real-Time PCR System (Applied Biosystems, USA). *OsActin1* (LOC_Os03g50885) served as an internal reference gene, and relative expression levels were calculated by using the 2^^–ΔΔCt^ method (Livak and Schmittgen, 2001). All assays were performed with three biological replicates, each with three technical replicates. Values were expressed as means **±** SE (n = 3). Different letters indicate significant differences among different groups, based on one-way ANOVA followed by Tukey’s multiple comparison test (*P* < 0.05). The primer sequences used in this study were listed in **Supplementary Table 1**.

### Yeast two-hybrid assays

Y2H assays were performed using the Matchmaker Yeast Two-Hybrid System (Clontech) according to the manufacturer’s instructions. The full-length coding sequence (CDS) of *IDR1* was cloned into the pGBKT7 vector as a bait, and the CDSs of selected overlapping DEGs were cloned into the pGADT7 vector as a prey. For each interaction test, a pair of bait and prey constructs (containing tested genes) were co-transformed into the *Saccharomyces cerevisiae* strain Y2HGold by using the lithium acetate-mediated method. The transformed yeast cells were initially grown on SD/-Leu/-Trp (SD-LT) deficient media at 30 °C for approximately 3 days to confirm successful co-transformation. Positive colonies were then transferred onto SD/-Leu/-Trp/-His (SD-LTH) deficient media and incubated at 30 °C for an additional 3–5 days to see protein-protein interactions. Images were captured after incubation by using a digital imaging system. The primer sequences used in this study were listed in **Supplementary Table 1**.

### Firefly Luminescence Complementation Imaging assays

LCI assays were performed in *Nicotiana benthamiana* leaves to check protein-protein interactions *in planta*. To do so, the full-length CDS of *IDR1* was fused to the N-terminal portion of *luciferase* gene (*nLUC*), while the CDSs of selected DEGs were separately fused to the C-terminal portion of *luciferase* gene (*cLUC*). The resulting constructs were paired, and they were introduced into *Agrobacterium tumefaciens* strain GV3101 and then co-infiltrated into fully expanded *N. benthamiana* leaves using standard agroinfiltration procedures. At 2–3 days post-infiltration, leaves were treated with 1 mM D-luciferin (Promega), followed by incubation in the dark for 7 min. Luminescence signals were subsequently detected using a Tanon 5200 chemiluminescence imaging system (Tanon, Shanghai, China). The well-established interaction pair SGT1a and RAR1 were included as a positive control, and the interaction pairs, each comprising one or two empty vectors, served as negative controls (Chen et al., 2008). The primer sequences used in this study were listed in **Supplementary Table 1**.

## Results

### *idr1–1* mutant seedlings exhibited enhanced tolerance to osmotic stress

**Figure 1:**
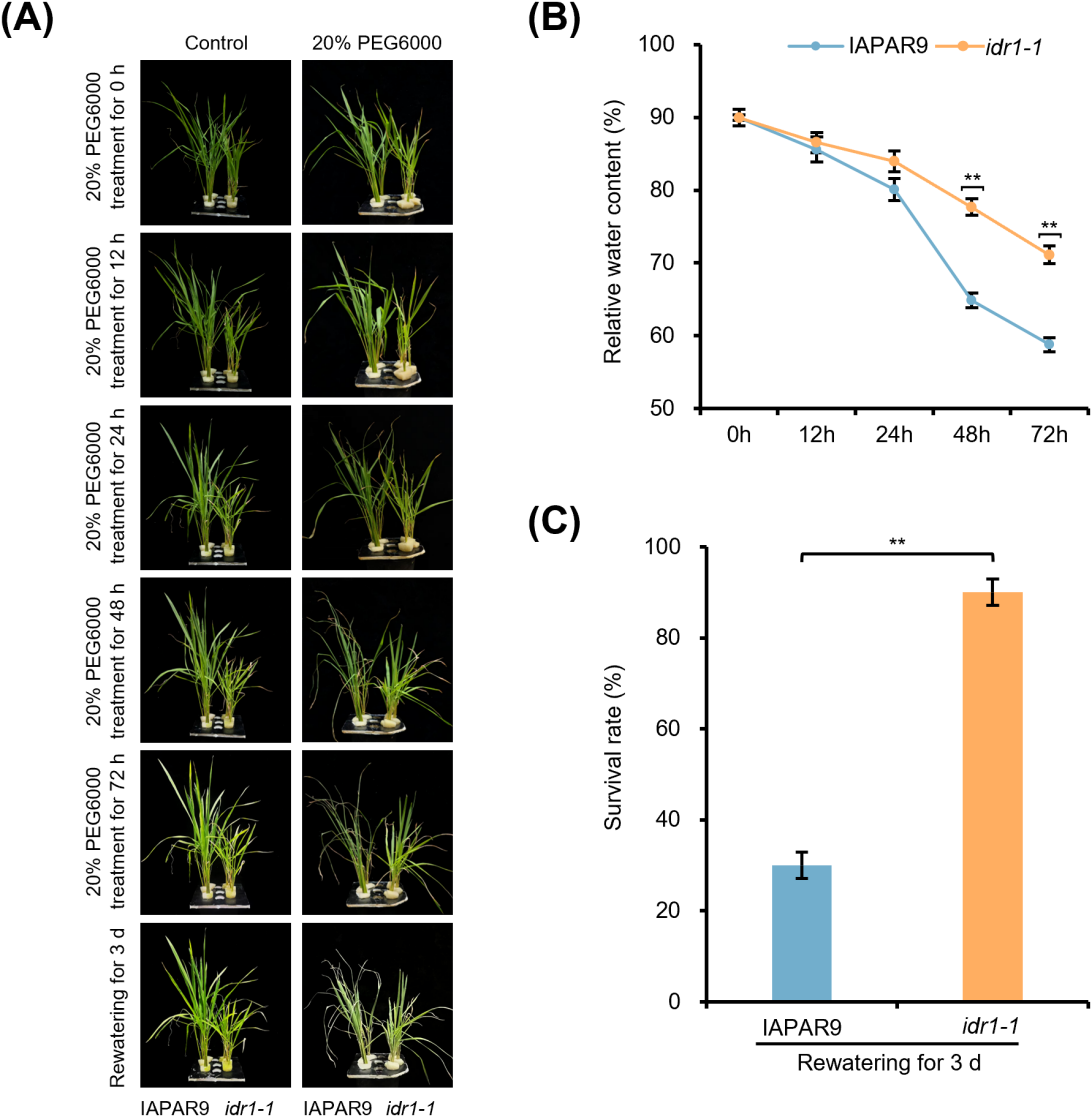
*idr1–1* mutant seedlings exhibited enhanced tolerance to 20% PEG-induced osmotic stress. **(A)** Representative images showing responses of *idr1–1* mutant and wild-type IAPAR9 seedlings to 20% PEG6000 treatments. Seedlings were photographed at the time points as indicated (0, 12, 24, 48, and 72 h) following PEG treatment and after rewatering for 3 days. **(B)** Changes of relative water contents of *idr1–1* mutant and wild-type IAPAR9 seedlings during 20% PEG treatments. **(C)** Survival rates of *idr1–1* mutant and wild-type IAPAR9 seedlings following PEG treatments and rewatering. Values are expressed as means ± SE of three independent experiments. Values in panels **(B, C)** are means ± SE from three biological replicates. Asterisks in panel **(B)** indicate significant differences between IAPAR9 and *idr1–1* samples at the same time points, based on two-way ANOVA followed by Šídák’s multiple comparison tests. Asterisks in panel **(C)** indicate significant differences between groups based on one-way ANOVA followed by Tukey’s multiple comparison tests. **P < 0.01.

To find out whether the *idr1–1* mutant plants exhibited altered tolerance to osmotic stress, we treated *idr1–1* mutant seedlings, along with wild-type IAPAR9 seedlings, with 20% PEG6000, and observed their responses over time, followed by survival assessment after rewatering (**Figure 1A**). Before PEG treatment, both the *idr1–1* mutant and wild-type IAPAR9 seedlings showed normal growth with no visible leaf rolling or wilting. After 12-h PEG treatment, leaves of the seedlings from both genotypes remained largely unchanged. After 24-h treatment, the wild-type IAPAR9 seedlings began to show slight leaf curling and drying at leaf tips, whereas the *idr1–1* seedlings showed no visible changes in leaf morphology. Following 48-h treatment, the IAPAR9 seedlings exhibited pronounced leaf rolling and stem yellowing, while the *idr1–1* seedlings only showed mild leaf curling and marginal yellowing at leaf tips. After 72-h treatment, the wild-type IAPAR9 seedlings displayed severe leaf wilting as well as yellowing and apparent stem yellowing, whereas the *idr1–1* seedlings were only affected moderately, with limited wilting and yellowing at leaf tips. To learn about physiological differences between *idr1–1* mutant and wild-type IAPAR9 seedlings, we measured relative water content under prolonged osmotic stress. The results showed that the relative water contents were decreased to a greater extent in the IAPAR9 seedlings than in the *idr1–1* mutant seedlings at the indicated time points (**Figure 1B**). After 3 days of rewatering, the *idr1–1* mutant seedlings almost completely recovered, whereas the wild-type IAPAR9 seedlings showed only a partial recovery. Statistics calculated from the control and PEG-treated seedlings revealed that, under the control (untreated) conditions, the survival rates of IAPAR9 and *idr1–1* mutant seedlings reached 100%, whereas after 3 days of rewatering following 72 h of PEG treatment, the survival rates decreased to ~30% for IAPAR9 seedlings and ~90% for *idr1–1* seedlings (**Figure 1C**). These results demonstrate that *idr1–1* mutant seedlings exhibit enhanced tolerance to PEG-induced osmotic stress, with reduced leaf water loss, less leaf damage, and higher survival rates, indicating that *idr1–1* mutation confers enhanced osmotic-stress tolerance during the seedling stage.

### Expression of stress-responsive genes induced by osmotic stress in *idr1–1* mutant seedlings

**Figure 2:**
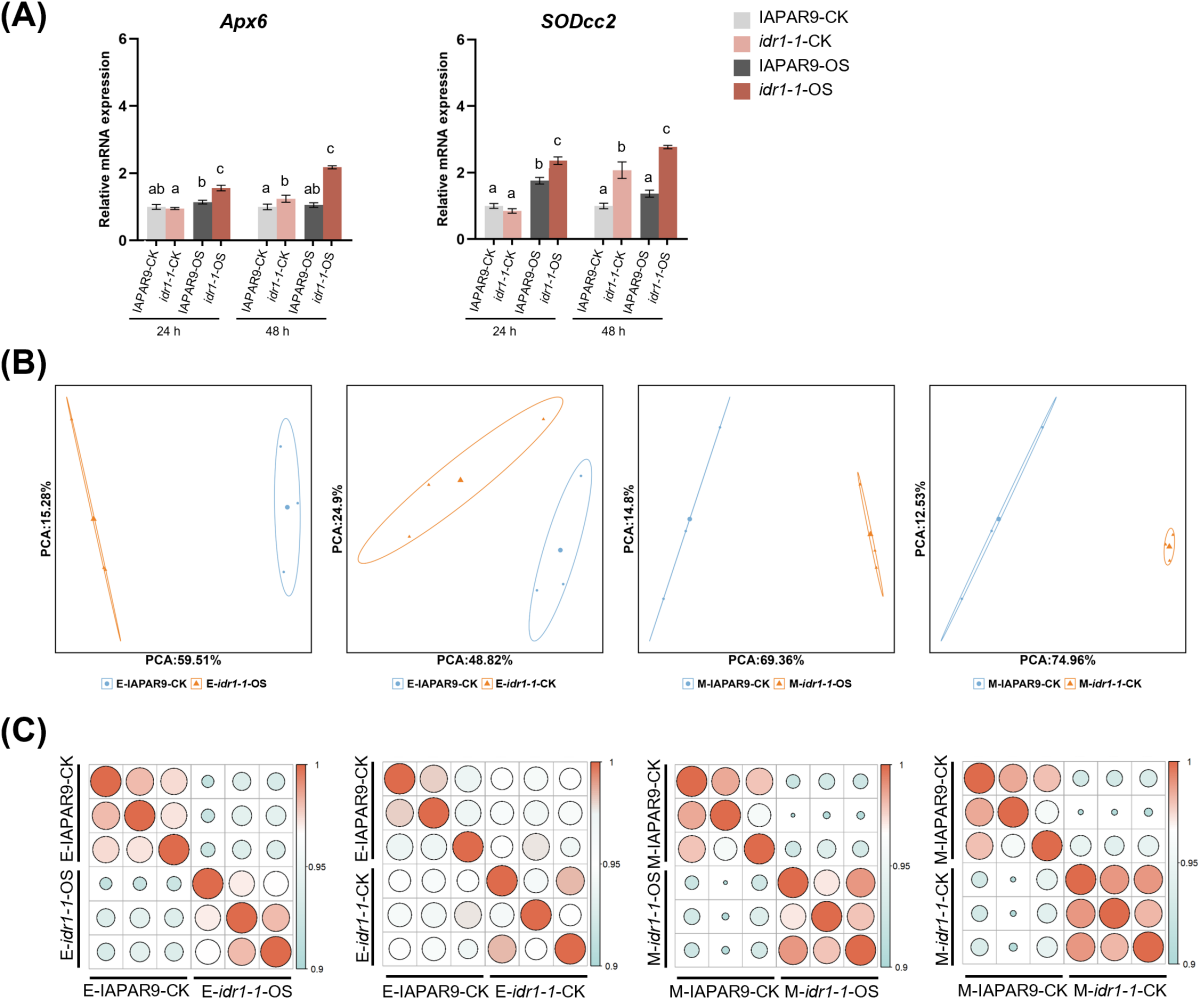
Examination of effectiveness of osmotic-stress treatments and quality of RNA-seq data derived from IAPAR9 and *idr1–1* mutant seedlings undergoing early or middle stage of osmotic stress. **(A)** Expression analyses of 2 osmotic stress-responsive genes (*Apx6* and *SODcc2*) in IAPAR9 or *idr1–1* mutant seedlings undergoing 24-h or 48-h osmotic stress. qRT-PCR data from each sample were normalized to *ACTIN*, and values are expressed as mean ± SE (n = 3) relative to their respective control samples (set to 1) in each treatment group. Different letters mark significant differences from the control samples in each treatment group (ANOVA, *P* < 0.05). **(B)** PCA of the mRNA-seq samples collected from IAPAR9 and *idr1–1* mutant seedlings under nontreatment or osmotic-stress treatment conditions. **(C)** Pairwise Pearson’s correlation analyses of mRNA-seq data sets as indicated.

To understand the underlying mechanisms for enhanced osmotic-stress resistance observed in *idr1–1* mutant seedlings at the transcriptional level, we decided to determine the transcriptomes of wild-type IAPAR9 and *idr1–1* mutant seedlings under both treatment conditions. To determine whether the osmotic-stress treatments were effective prior to sampling, two genes reportedly involved in stress responses were chosen and used for qRT-PCR assays. The results indicated that following the 24-h PEG treatments, *Apx6* expression in IAPAR9 seedlings was slightly induced (see IAPAR9-OS), but the differences did not reach statistical significance when compared to untreated IAPAR9 seedlings (see IAPAR9-CK); however, the treatments induced a significant upregulation of *SODcc2* in the IAPAR9 seedlings (see IAPAR9-OS) (**Figure 2A**). For *idr1–1* seedlings, the 24-h PEG treatments led to a significant induction of *Apx6* and *SODcc2* expression (see *idr1-1*-OS) in comparison to the expression in untreated *idr1–1* mutant seedlings (see *idr1-1*-CK) (**Figure 2A**). This suggests that 24-h PEG treatments were sufficient to create osmotic stress to induce upregulation of some endogenous stress-responsive genes, especially for the genes in the *idr1–1* mutant background. Under conditions of 48-h PEG treatments, although *Apx6* and *SODcc2* expression were marginally induced and no significant differences in expression levels between untreated IAPAR9 seedlings (see IAPAR9-CK) and PEG-treated IAPAR9 seedlings (see IAPAR9-OS) were observed, their expression levels were significantly induced in the *idr1–1* mutant seedlings after the PEG treatments (see *idr1-1*-OS) when compared with those in the untreated *idr1–1* mutant seedlings (see *idr1-1*-CK) (**Figure 2A**). It is worth noting that the degrees of upregulation of *Apx6* and *SODcc2* in *idr1–1* mutant seedlings after 48-h PEG treatment were greater than those observed after 24-h PEG treatment. Therefore, the responses of both genotypes to 24-h and 48-h PEG treatment were rated as the early-stage response (designated by E) and the middle-stage response (designated by M), respectively. To determine the transcriptomic profiles of IAPAR9 and *idr1–1* mutant seedlings under control (untreated) and different osmotic stress conditions, leaves of IAPAR9 and *idr1–1* mutant seedlings undergoing no treatment (control) or 24-h/48-h PEG treatments were collected and the corresponding mRNAs were subjected to mRNA sequencing (mRNA-seq). To assess the quality and consistency of these RNA-seq data, principal component analysis (PCA) and sample correlation analyses were performed afterward. PCA revealed an overall consistency among the three biological replicates of IAPAR9 or *idr1–1* mutant samples under control or osmotic stress conditions, because they tended to cluster together (**Figure 2B**). Besides, there was clear separation between samples, suggesting that different samples exhibited quite distinct expression profiles in general (**Figure 2B**; **Supplementary Figure 1A**). Consistently, pairwise Pearson’s correlation analyses showed high correlation among these biological replicates across all genotypes (**Figure 2C**; **Supplementary Figure 1B**), further supporting the reliability of PCA analyses. Thus, such mRNA-seq data were appropriate for further analysis to reveal the transcriptional differences between wild-type IAPAR9 and *idr1–1* mutant seedlings under PEG-induced osmotic stress conditions.

### Transcriptomic changes underlying enhanced osmotic-stress tolerance in *idr1–1* mutant seedlings

**Figure 3:**
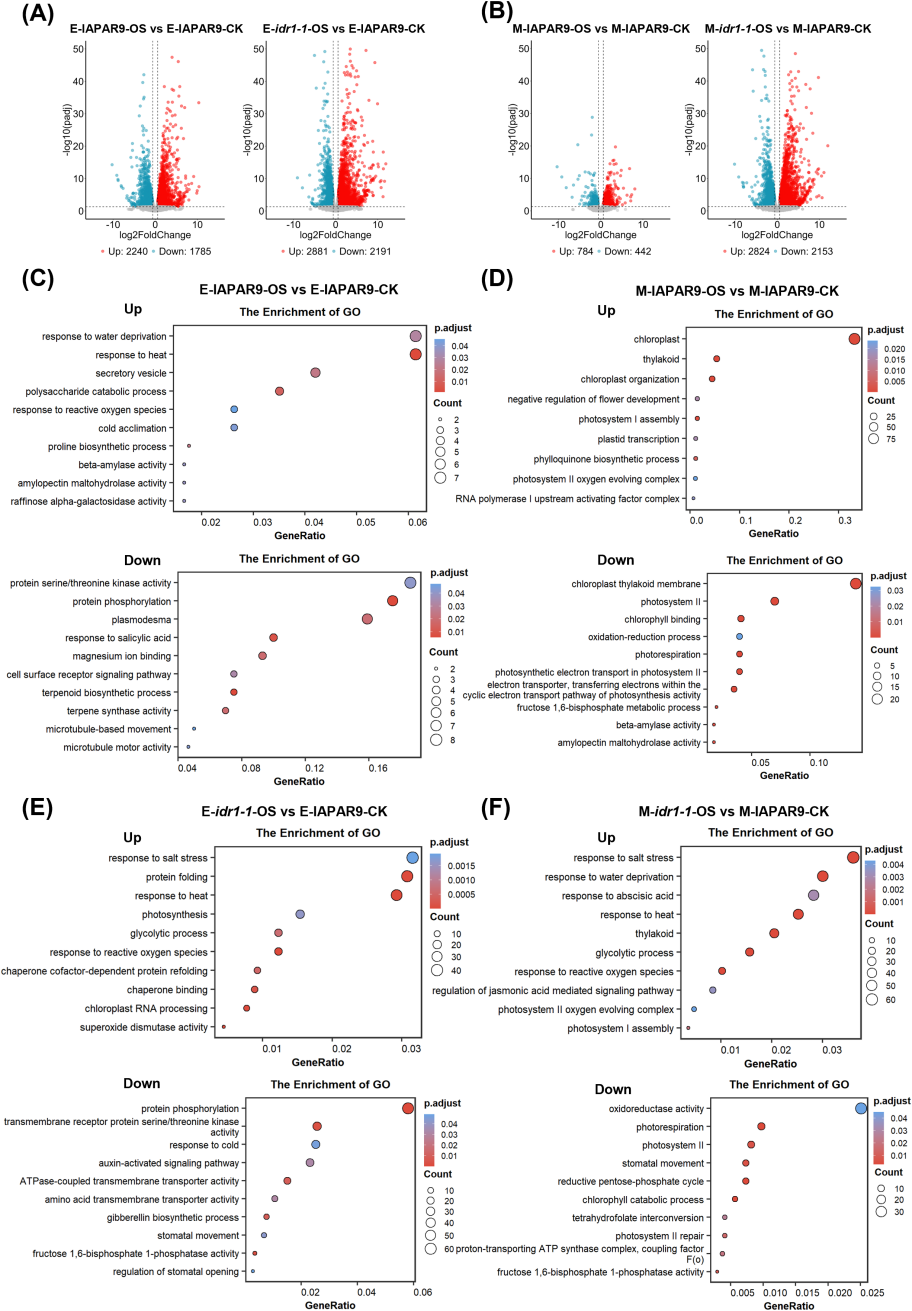
Identification of upregulated or downregulated DEGs under osmotic stress conditions and GO term analyses of the DEGs. **(A, B)** Volcano plots showing DEGs identified from IAPAR9 or *idr1–1* mutant seedlings undergoing early **(A)** or middle **(B)** stage of osmotic stress. Red and blue dots indicate significantly upregulated and downregulated DEGs, respectively, while gray dots denote non-significant DEGs. Dashed vertical and horizontal lines indicate thresholds to judge statistical significance (false discovery rate (FDR) < 0.05 and |Fold Change| ≥ 1.5). The numbers of up- and downregulated DEGs are given below each plot. **(C, D)** Gene Ontology (GO) enrichment analyses of DEGs coming from comparison group E-IAPAR9-OS vs E-IAPAR9-CK in panels **(A, C)**, and from comparison group M-IAPAR9-OS vs M-IAPAR9-CK in panels **(B, D)**. **(E, F)** GO enrichment analyses of DEGs coming from comparison group E-*idr1-1*-OS vs E-IAPAR9-CK in panels **(A, E)**, and from comparison group M-*idr1-1*-OS vs M-IAPAR9-CK in panels **(B, F)**. For panels **(C–F)**, dot sizes are proportional to the numbers of DEGs in the GO terms, and the color key indicates the adjusted P value for enrichment.

To obtain an overall picture of transcriptional changes associated with osmotic stress for *idr1–1* mutant seedlings, differential gene expression analyses were performed using those mRNA-seq data. The results showed that at the early stage of osmotic stress, 2240 upregulated and 1785 downregulated DEGs were identified in wild-type IAPAR9 seedlings (E-IAPAR9-OS) relative to those under control conditions (E-IAPAR9-CK) (**Figure 3A**, left panel). The early-stage osmotic stress also induced a large number of DEGs in *idr1–1* mutant seedlings (E-*idr1-1*-OS) relative to IAPAR9 seedlings without undergoing the osmotic-stress treatment (E-IAPAR9-CK): 2881 upregulated and 2191 downregulated (**Figure 3A**, right panel). This suggests that the numbers of DEGs induced by osmotic stress in wild-type IAPAR9 and *idr1–1* mutant seedlings at the early stage of osmotic stress were all quite large. At the middle stage of osmotic stress, 784 upregulated and 442 downregulated DEGs were detected in wild-type IAPAR9 seedlings (M-IAPAR9-OS) relative to those under corresponding control conditions (M-IAPAR9-CK) at the middle stage of osmotic stress (**Figure 3B**, left panel). By comparison, 2824 upregulated and 2153 downregulated DEGs were identified in the *idr1–1* mutant seedlings (**Figure 3B**, right panel), the DEG numbers similar to that observed in the *idr1–1* mutant seedlings undergoing early-stage osmotic stress (**Figure 3A**, right panel). It is worth noting that for the wild-type IAPAR9 seedlings, the numbers of DEGs gradually decreased markedly from early to middle stage; by contrast, for the *idr1–1* mutant seedlings, the numbers of DEGs were almost unchanged (**Figures 3A, B**), suggesting that *idr1–1* mutation contributes to persistent expression of DEGs as osmotic stress progressed. By comparison, only 202 upregulated and 57 downregulated DEGs were identified under early-stage control conditions (E-*idr1-1*-CK), and 437 upregulated and 282 downregulated DEGs detected under middle-stage control conditions (M-*idr1-1*-CK) for *idr1–1* mutant seedlings relative to wild-type IAPAR9 seedlings (E-IAPAR9-CK and M-IAPAR9-CK) (**Supplementary Figures 2A, B**), suggesting that *idr1–1* mutation only induced expression of a relatively small number of DEGs under the control conditions. Interestingly, the numbers of upregulated (437) and downregulated (282) DEGs detected under middle-stage control conditions were markedly greater than those of upregulated (202) and downregulated (57) DEGs detected under early-stage control conditions (**Supplementary Figures 2A, B**), suggesting that *idr1–1* mutation had triggered a progressive increase in the numbers of DEGs as the seedlings developed. (All DEGs derived from all stages are listed in **Supplementary Dataset S1**).

To further understand the functional implications of these above-mentioned transcriptional alterations, Gene Ontology (GO) enrichment analyses were performed to identify the associated biological processes in which these DEGs were involved. For the comparison group E-IAPAR9-OS vs E-IAPAR9-CK, the DEGs fell into more than 10 GO term categories, and the top 10 categories were selected for further analysis (**Figure 3C**). For the upregulated DEGs, the enriched GO terms included responses to water deprivation and heat, response to reactive oxygen species (ROS), proline biosynthetic process, raffinose alpha-galactosidase activity and so on, which were all known to be related to responses to osmotic stress (**Figure 3C**, upper panel). For the downregulated DEGs, the enriched GO terms were associated with the following processes: protein serine/threonine kinase activity, protein phosphorylation, response to salicylic acid, cell surface receptor signaling pathway, terpenoid biosynthesis-related processes, microtubule-related movement, and so on (**Figure 3C**, lower panel). These results demonstrate that in wild-type IAPAR9 seedlings, both upregulated and downregulated DEGs were clearly associated with water deficiency-induced stress responses, ROS homeostasis, production of osmoprotectants, translational modification of proteins, signal transduction, etc. For the upregulated DEGs from the comparison group E-*idr1-1*-OS vs E-IAPAR9-CK, the enriched GO terms were basically similar to the upregulated DEGs found in the comparison group E-IAPAR9-OS vs E-IAPAR9-CK (**Figure 3E**, upper panel), suggesting that *idr1–1* mutation does not cause considerable changes in classifications of DEGs derived from wild-type IAPAR9 seedlings under the early-stage osmotic stress. By comparison, for the downregulated DEGs, the enriched GO terms from the comparison group E-*idr1-1*-OS vs E-IAPAR9-CK were quite different from those coming from the comparison group E-IAPAR9-OS vs E-IAPAR9-CK (**Figure 3E**, lower panel), that is, there were several GO terms, such as hormone-related responses/pathways (auxin and GA), regulation of stomatal actions, and response to cold, which were not found in the GO terms from the comparison group E-IAPAR9-OS vs E-IAPAR9-CK (**Figure 3E**, lower panel). This demonstrates that *idr1–1* mutation gave rise to a large number of new classifications of downregulated genes at the early stage of osmotic stress. For the upregulated DEGs from the comparison group M-IAPAR9-OS vs M-IAPAR9-CK, the enriched GO terms were associated with chloroplast, thylakoid, chloroplast organization, photosystem I assembly, photosystem II oxygen evolving complex, and so on, which were mainly concentrated on alterations of photosynthetic systems (**Figure 3D**, upper panel). Similarly, for the downregulated DEGs, the enriched GO terms were also mainly connected with changes of photosynthetic systems, oxidation-reduction process, adjustment of antioxidation, etc. (**Figure 3D**, lower panel). Thus, this indicates that the wild-type IAPAR9 seedlings coped with increased osmotic stress mainly by modulating the photosynthetic processes and their antioxidant capacity. For the upregulated DEGs from the comparison group M-*idr1-1*-OS vs M-IAPAR9-CK, the enriched GO terms were associated with responses to salt, water deprivation and heat, response to ABA, thylakoid, response to ROS, regulation of jasmonic acid (JA)-mediated signaling pathway, photosystem II oxygen evolving complex, photosystem I assembly, and so on (**Figure 3F**, upper panel). Likewise, for the downregulated DEGs, the enriched GO terms were, at least in part, similar to those found in the upregulated DEGs, with a few new classifications, such as oxidoreductase activity, photorespiration, and adjustment of stomatal actions (**Figure 3F**, lower panel). Collectively, the enriched GO terms induced by *idr1–1* mutation appear to be concentrated on alterations of photosynthetic systems, responses to water deficiency, heat as well as phytohormones (ABA, JA and GA), alterations of antioxidation process, regulation of stomatal actions, which are somewhat different from those found in the wild-type IAPAR9 seedlings under the same treatment conditions. In addition, GO analysis of another comparison group E-*idr1-1*-CK vs E-IAPAR9-CK revealed a significant enrichment of DEGs associated with photosynthetic system, response to ROS, stomatal movement, metal ion transport, etc. (**Supplementary Figure 2C**). Moreover, GO analysis of another comparison group M-*idr1-1*-CK vs M-IAPAR9-CK showed an enrichment of DEGs related to responses to ABA and JA, responses to water deprivation and heat, oxidoreductase activity, regulation of photosynthetic systems, protein post-translational modifications, etc. (**Supplementary Figure 2D**). Taken together, the enhanced tolerance of *idr1–1* mutant seedlings to osmotic stress may stem from marked changes in DEG profiles at the early and middle stages of osmotic stress, especially at the middle stage, when compared to those from wild-type IAPAR9 seedlings, and these changes may strengthen the capacity of *idr1–1* mutant seedlings to cope better with severe osmotic stress.

### DEGs co-regulated by *idr1–1* mutation and osmotic stress were overrepresented by GO terms related to photosynthetic systems

**Figure 4:**
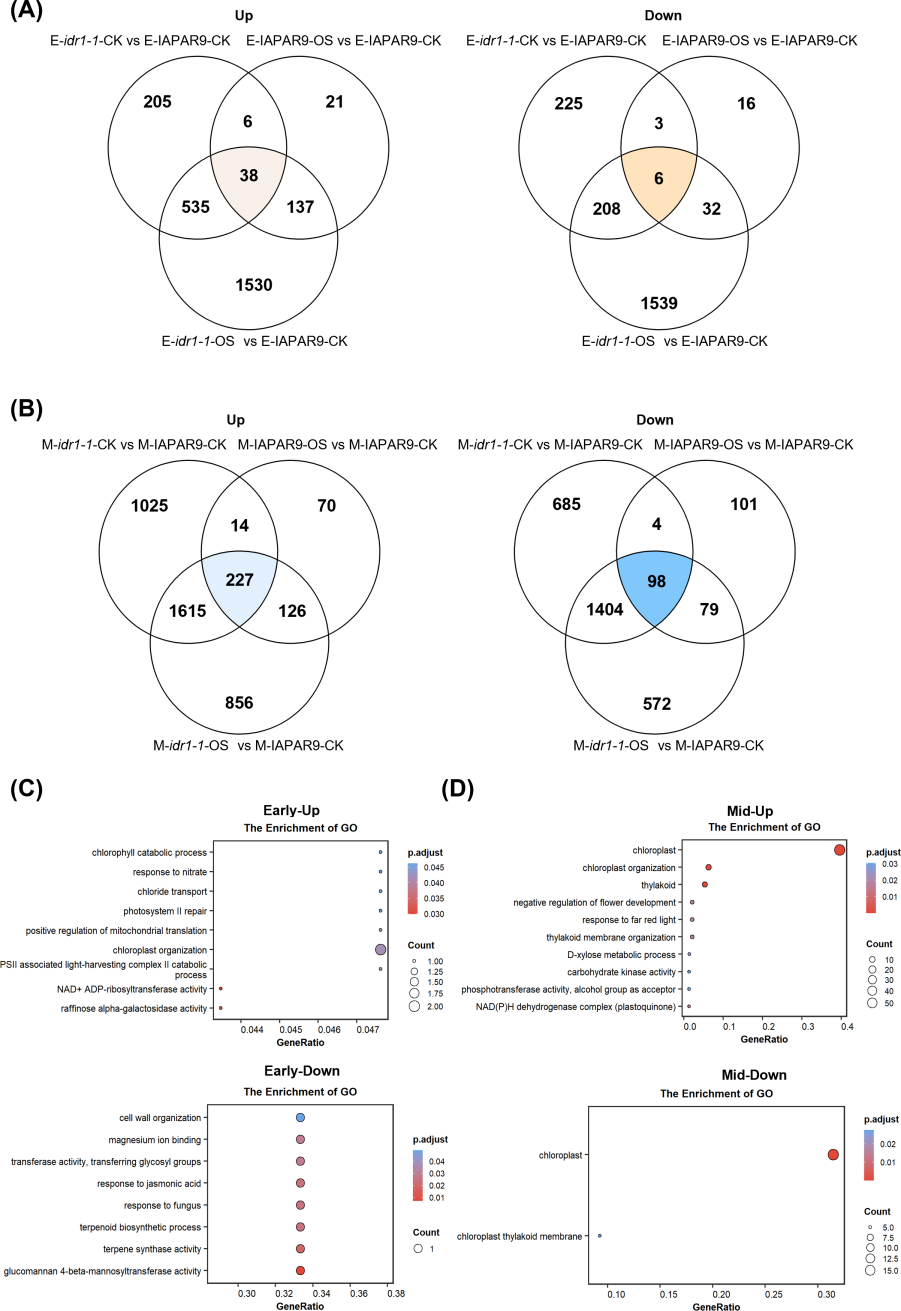
Identification of DEGs that were co-regulated by *idr1–1* mutation and osmotic stress at early or middle stages of osmotic stress and GO term analyses of the DEGs. **(A)** Venn diagrams showing the overlap of upregulated (left panel) and downregulated (right panel) DEGs following the early-stage osmotic stress. **(B)** Venn diagrams showing the overlap of upregulated (left panel) and downregulated (right panel) DEGs following the middle-stage osmotic stress. The numerals in each sector are the numbers of overlapping DEGs. **(C)** GO enrichment analyses for overlapping upregulated (Early-Up) and downregulated (Early-Down) DEGs from A at the early stage of osmotic stress. **(D)** GO enrichment analyses for overlapping upregulated (Mid-Up) and downregulated (Mid-Down) DEGs from B at the middle stage of osmotic stress. For panels **(C, D)**, dot sizes are proportional to the numbers of DEGs in the GO terms, and the color key indicates the adjusted *P* value for enrichment.

To further identify genes co-regulated by the *idr1–1* mutation and osmotic stress for better understanding the mechanisms behind the enhanced osmotic-stress tolerance observed in *idr1–1* mutant seedlings, overlap analyses were performed among three sets of DEGs at both osmotic-stress stages. We first conducted overlap analyses between the DEGs from two comparison groups: E-*idr1-1*-CK vs E-IAPAR9-CK and E-IAPAR9-OS vs E-IAPAR9-CK, to obtain overlapping DEGs that were induced by both *idr1–1* mutation and osmotic stress. Subsequently, the overlapping DEGs were further compared with the DEGs from the comparison group E-*idr1-1*-OS vs E-IAPAR9-CK, which included the genes induced by *idr1–1* mutation and/or osmotic stress, to further narrow down the list of those DEGs induced simultaneously by *idr1–1* mutation and osmotic stress. The corresponding gene lists of upregulated and downregulated overlapping DEGs identified at each stage of osmotic stress were shown in **Supplementary Dataset S2**. By using this method, we obtained a limited number of DEGs commonly shared among all three DEG sets, including 38 upregulated and 6 downregulated genes at the early stage of osmotic stress (**Figure 4A**). These results indicate that the number of genes affected by both osmotic stress and *idr1–1* mutation is quite small when osmotic stress occurred at the early stage. In contrast, a substantially larger number of DEGs common to the three DEG sets were identified at the middle stage of osmotic stress, including 227 upregulated and 98 downregulated genes (**Figure 4B**), suggesting that as osmotic stress progressed, more genes were induced by both *idr1–1* mutation and osmotic stress. These results are consistent with the observations that *idr1–1* displays elevated osmotic tolerance at the middle stage of osmotic stress than at the early stage, suggesting that *idr1–1* mutation-induced transcriptional activation occurred gradually as osmotic stress progressed.

GO term analyses of the overlapping DEGs derived from early-stage osmotic stress revealed that the enriched GO terms were mainly associated with photosynthetic systems (for upregulated DEGs) and secondary metabolism as well as responses to hormones (for downregulated DEGs), including chlorophyll catabolic process, photosystem II repair, chloroplast organization, PSII-associated light-harvesting complex II catabolic process, cell-wall organization, response to JA, terpenoid biosynthesis-related processes, etc. (**Figure 4C**). The overlapping upregulated and downregulated DEGs derived from middle-stage osmotic stress were enriched in a total of 12 GO terms, including the processes associated with photosynthetic systems, response to far-red light, D-xylose metabolic process, carbohydrate kinase activity, NAD(P)H dehydrogenase complex, etc. (**Figure 4D**). Collectively, these results provide a clear demonstration that *idr1–1* mutation is able to largely alter the activities of photosynthetic systems at the early stage of osmotic stress, and these changes persisted until the middle stage; in addition, *idr1–1* mutation also led to alterations of some secondary metabolisms, oxidation-reduction processes and hormone responses. Thus, it is very likely that the enhanced resistance to osmotic stress stems from these transcriptomic changes.

### Expression changes of DEGs co-regulated by *idr1–1* mutation and osmotic stress in *idr1-1*

**Figure 5:**
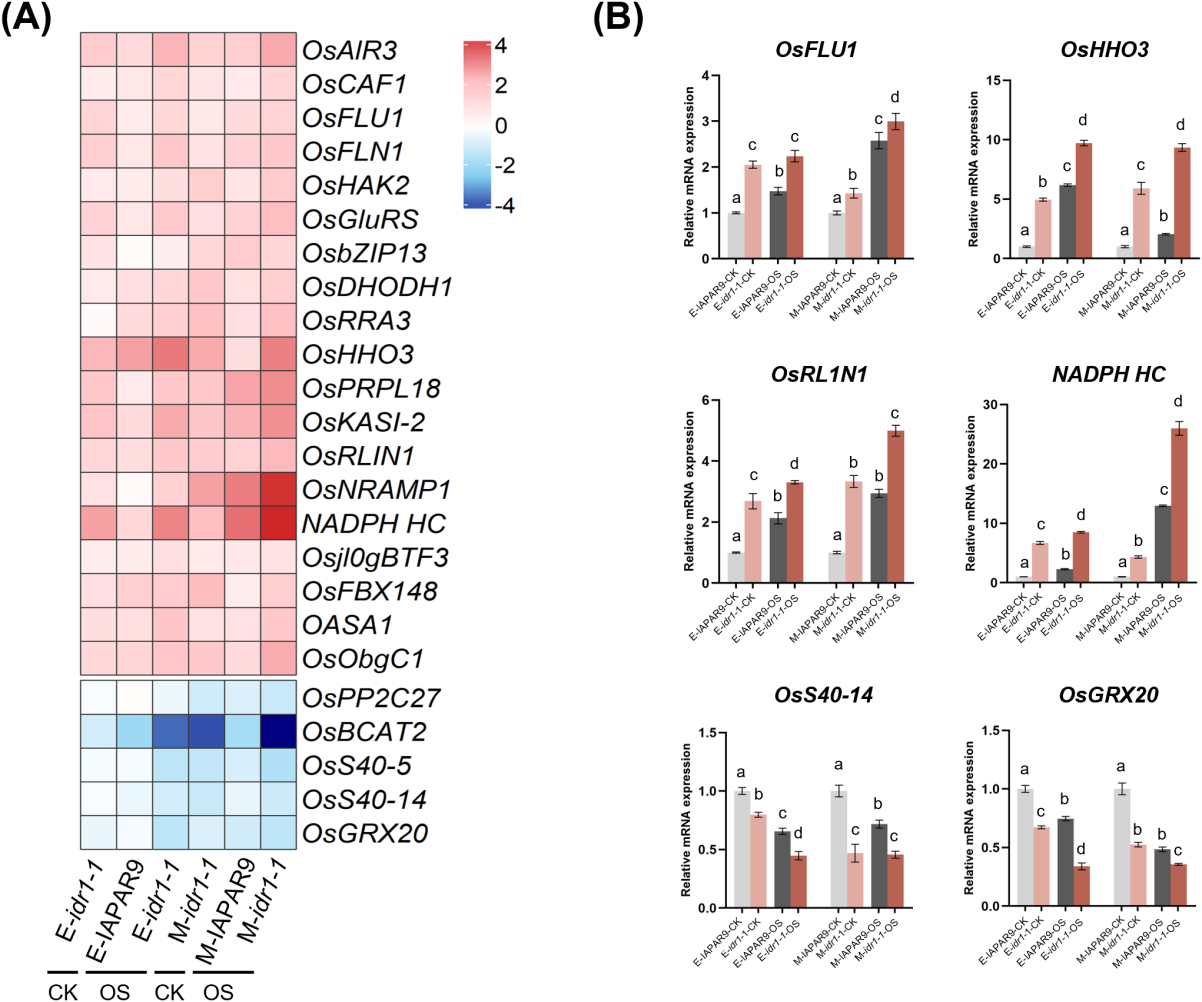
Detection of expression of 24 selected DEGs that were co-regulated by *idr1–1* mutation and osmotic stress. **(A)** Heatmaps showing the expression profiles of 24 selected DEGs in *idr1–1* mutant seedlings that were co-regulated by *idr1–1* mutation and osmotic stress. The color key represents log_2_-transformed fold changes. CK, no treatment; OS, undergoing the early or middle stage of osmotic stress. **(B)** Validation of expression levels of 6 selected DEGs from panel **(A)** by qRT-PCR assays. The expression levels of 6 representative upregulated or downregulated DEGs were examined in leaf samples collected from the seedlings undergoing osmotic stress at early or middle stage of PEG treatments. qRT-PCR data from each sample were normalized to those of *ACTIN*, and values are expressed as mean ± SE (n = 3) relative to their respective control samples (set to 1) in each treatment group. Different letters mark significant differences from the control samples in each treatment group (ANOVA, *P* < 0.05).

To further find out which genes out of the above-mentioned overlapping gene sets are involved in *idr1–1* mutation-induced resistance to osmotic stress, we chose a dozen DEGs and examined their expression levels. Heatmap analyses of these genes showed that at the early stage of osmotic stress, most of these DEGs coming from *idr1–1* mutant seedlings exhibited higher or lower expression levels to varying extents compared with those from IAPAR9 seedlings (see the 2nd and 3rd columns in **Figure 5A**); besides, the majority of the examined DEGs coming from *idr1–1* mutant seedlings also showed higher or lower expression levels in comparison with those in *idr1–1* mutant seedlings that did not undergo osmotic stress (see the 3rd and 1st columns in **Figure 5A**). A similar situation was observed for the *idr1–1* mutant and IAPAR9 seedlings at the middle stage of osmotic stress (see the 4th to 6th columns in **Figure 5A**).

In order to experimentally confirm the expression levels of these genes, a total of 6 genes, *OsFLU1*, *OsHHO3*, *OsRLIN1*, *NADPH HC*, *OsS40–14* and *OsGRX20*, were selected for qRT-PCR assays. The results demonstrated that in *idr1–1* mutant seedlings, 4 genes, i.e. *OsFLU1*, *OsHHO3*, *OsRLIN1* and *NADPH HC*, were all significantly upregulated, while 2 genes, i.e. *OsS40–14* and *OsGRX20*, were markedly downregulated, when compared to IAPAR9 seedlings, under control or osmotic stress conditions at both stages (**Figure 5B**). It is evident that osmotic-stress treatment induced stronger upregulation or downregulation of these genes compared with the control treatment, suggesting that these genes are all osmotic stress-inducible genes (**Figure 5B**). *OsFLU1*, a rice homolog of *Arabidopsis FLU*, encodes a negative regulator of protochlorophyllide synthesis that helps prevent photooxidative damage during dark-to-light transitions (Li et al., 2019); therefore, the upregulation of *OsFLU1* in *idr1–1* mutant seedlings under osmotic stress may favor the prevention of photooxidative damage caused by such a stress. OsHHO3, OsRLIN1 and NADPH HC were reported to be involved in ROS scavenging and chloroplast functions, and their upregulation induced by osmotic stress in *idr1–1* mutant seedlings may contribute to eliminating excessive ROS and proper functioning of chloroplasts. OsHHO3 belongs to the NIGT1/HHO family of transcription factors and functions as a component of red-light and ABA signaling pathways, and it was reported to modulate stomatal aperture in response to external stress (Li et al., 2022). In addition, OsHHO3 was also discovered to be able to form a transcriptional complex with OsPIL15 to activate *OsABI5* expression, thereby influencing stomatal movement and transpiration (Li et al., 2022). *OsRLIN1*, encoding a putative coproporphyrinogen III oxidase involved in tetrapyrrole biosynthesis, had been reported to mediate light-dependent lesion formation via excessive ROS accumulation (Sun et al., 2011). NADPH HC reportedly functions in electron transfer and antioxidant regeneration, suggesting a potential impact on plant physiological processes and stress tolerance (Islam et al., 2024). *OsS40–14* and *OsGRX20* were both transcriptionally repressed in *idr1–1* seedlings as shown by mRNA-seq and qRT-PCR results (**Figure 5**). *OsS40–14* encodes a nucleus-localized senescence regulator that integrates multiple hormone signaling pathways, including ABA, SA, and MeJA (Habiba et al., 2021). Thus, its repression in *idr1–1* mutant seedlings may contribute to the attenuation of ABA-mediated senescence signaling, which may help delay stress-induced leaf aging and sustain physiological activities of leaves under osmotic stress (Zheng et al., 2019). OsGRX20, a CPYC-type glutaredoxin, functions in maintaining cellular redox balance through glutathione-dependent ROS scavenging and regulation of antioxidant enzymes (Ning et al., 2018); hence, its lowered expression in *idr1–1* mutant seedlings may reflect a reduced oxidative load or more stable intracellular redox state under osmotic stress conditions.

### Interactions of proteins encoded by DEGs co-regulated by *idr1–1* mutation and osmotic stress with IDR1

**Figure 6:**
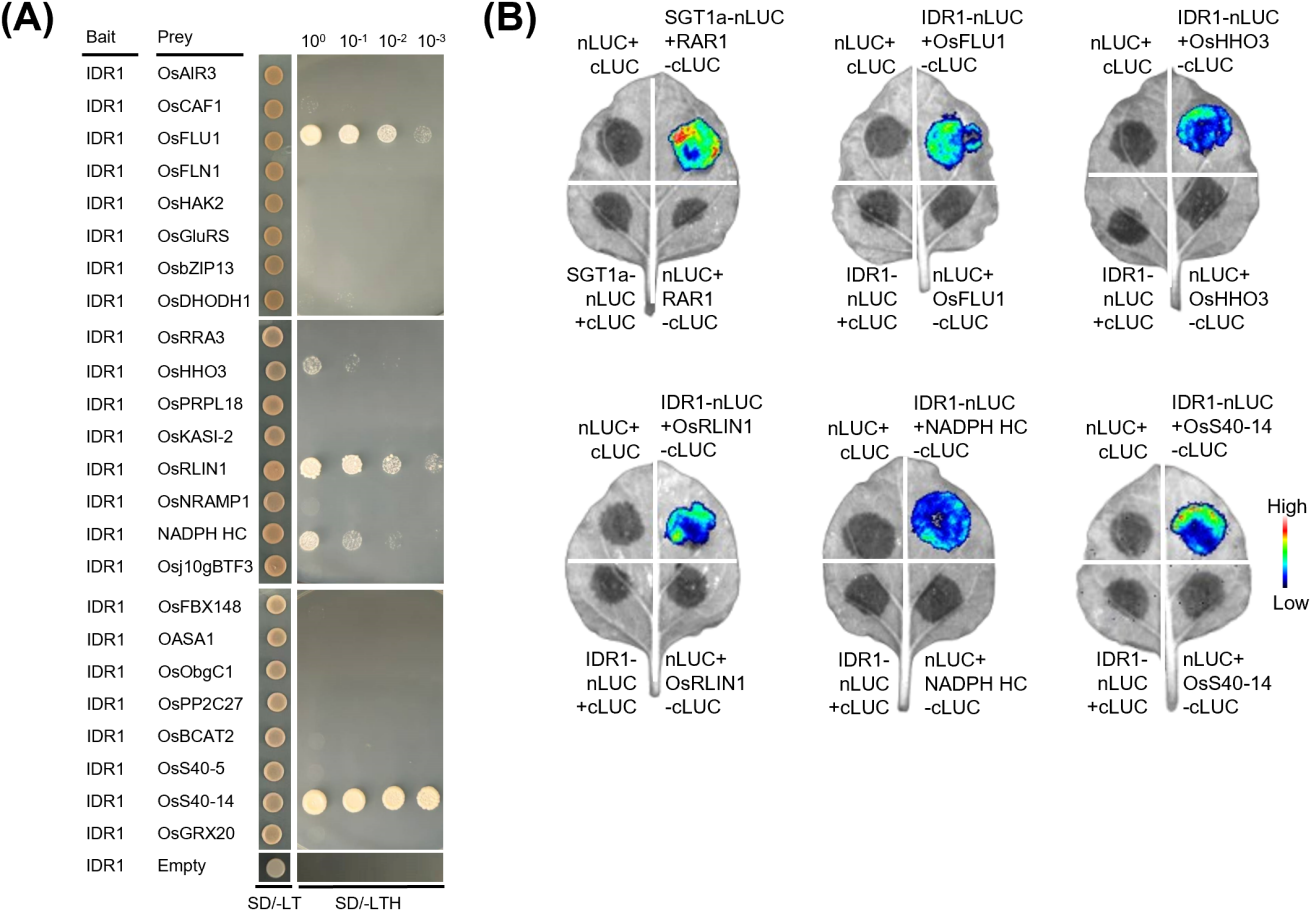
Assays for interactions between IDR1 and a collection of proteins encoded by the genes simultaneously induced by *idr1–1* mutation and osmotic stress. **(A)** Tests of interactions between IDR1 and each of the 24 proteins as shown in **Figure 5A** by Y2H assays, which were co-regulated by *idr1–1* mutation and osmotic stress. Yeast cells co-transformed with construct pairs as indicated were grown on selective media lacking leucine, tryptophan, and histidine. **(B)** Confirmation of interactions between IDR1 and the 5 identified proteins (OsFLU1, OsHHO3, OsRLIN1, NADPH HC, and OsS40-14) by LCI assays. The pair of SGT1a-nLUC and RAR1-cLUC served as a positive control to show the success of agroinfiltration.

Because of the fact that the product of a gene transcriptionally regulated by a regulatory protein can interact with such a regulatory protein, to ascertain whether the proteins encoded by those DEGs shown in **Figure 5A** (they were transcriptionally regulated by IDR1) are able to interact with IDR1, yeast two-hybrid (Y2H) assays were conducted between IDR1 and each of the proteins derived from these DEGs. The results demonstrated that of the 24 proteins tested, five proteins, including OsFLU1, OsHHO3, OsRLIN1, NADPH HC, and OsS40-14, were found to interact with IDR1 (**Figure 6A**). These interactions were further confirmed by Firefly Luminescence Complementation Imaging (LCI) assays (**Figure 6B**). *OsFLU1* encodes a negative regulator of protochlorophyllide synthesis (Li et al., 2019), and its interaction with IDR1 suggests that IDR1 may be involved in regulating chlorophyll biosynthesis and oxidative stress responses under osmotic stress conditions, which probably explains why the *idr1–1* mutant plants have dark-green leaves. *OsHHO3* encodes a NIGT1/HHO-family transcription factor functioning in red-light and ABA signaling (Li et al., 2022), and the interaction between IDR1 and OsHHO3 implies that IDR1 may participate in crosstalk between G protein and ABA-mediated regulatory pathways under osmotic stress, which may also be one of the reasons why *idr1–1* mutant plants exhibit enhanced drought tolerance (Zu et al., 2021). *OsRLIN1*, a tetrapyrrole pathway gene encoding a coproporphyrinogen III oxidase, mediates light-induced ROS accumulation (Sun et al., 2011); the IDR1-OsRLIN1 interaction suggests that G-protein signaling may be involved in chloroplast-derived oxidative pathways and ROS homeostasis under osmotic stress. NADPH HC functions in electron transfer and antioxidant regeneration, contributing to stress tolerance (Islam et al., 2024); therefore, the interaction between NADPH HC and IDR1 possibly enhances antioxidative capacity of the seedlings under osmotic stress. OsS40-14, a nucleus-localized transcriptional activator, promotes leaf senescence and regulates carbon allocation (Habiba et al., 2021). Thus, the interaction of OsS40–14 with IDR1 implies that IDR1 may help suppress senescence-associated pathways and delay the progression of osmotic stress-induced leaf aging. Taken together, these results indicate that there might be extensive crosstalk between the G-protein pathway and several important pathways/processes, such as protochlorophyllide biosynthesis, ABA-mediated regulatory pathway, chloroplast-derived oxidative pathway, ROS homeostasis and senescence-associated pathway, ultimately leading to enhanced tolerance to osmotic stress observed in the *idr1–1* mutant seedlings.

## Discussion

Tolerance of rice to water deficit is a complex trait that depends on coordinated changes in physiology, gene expression, metabolism, and so on (Zargar et al., 2021). Similar to the previous study (Zu et al., 2021), in this study the *idr1–1* mutant was also found to exhibit enhanced osmotic tolerance at the early and middle stages of osmotic stress. This tolerance performance provides a clear demonstration that IDR1 functions as a negative regulator of drought tolerance as reported previously and of PEG-induced osmotic-stress tolerance shown in this study. Thus, this study lays the groundwork for better understanding the roles of IDR1 in coping with osmotic stress.

Since the discovery of Gα protein in plant species, many studies centering around the biological functions of Gα protein had been conducted. Rice plants carrying a null *Gα* gene exhibited very noticeable developmental phenotypes, including reduced plant heights and shortened panicle as well as grain lengths, suggesting that Gα is closely associated with plant organ development (Ashikari et al., 1999). Subsequent investigations revealed that Gα formed a heterotrimer with Gβγ subunits and appeared to be involved in different signaling pathways, such as GA- and BR-mediated signaling transduction. It had been shown that two GA-induced genes, *Ramy1A* and *GAmyb*, displayed very significantly decreased expression levels in *d1* mutant (a null mutant of *Gα* gene), suggesting that Gα subunit is required for the transcription of both the genes (Ueguchi-Tanaka et al., 2000). Another study demonstrated that Gα subunit was able to interact with TUD1, which participated in the BR signaling pathway; therefore, *d1* mutant plants displayed similar dwarf stature and shortened internodes as *tud1* mutant plants did (Hu et al., 2013). So, Gα subunit appears to be essential for normal development of plant stature and organ morphologies in rice. Apart from the roles of Gα subunit in plant development, Gα subunit was also discovered to play a part in stress tolerance. Loss-of-function mutation of *Gα* gene in rice attenuated growth inhibition and cell senescence caused by NaCl treatment (Urano et al., 2014). *d1* mutant was found to be highly resistant to ethylene and H_2_O_2_-induced cell death occurring for root primordia; transcriptomic analysis revealed that ethylene and H_2_O_2_ treatments changed gene expression of epidermal cells undergoing cell death, but these expression changes did not happen to a large extent in *d1* mutant plants, which may be the reason why *d1* mutation is able to prevent epidermal cells from cell death (Steffens and Sauter, 2009). *COLD1* encodes a regulator of G-protein signaling, and overexpression of *COLD1*^jap^ natural allele significantly enhances chilling tolerance, whereas downregulation of such an allele renders plants sensitive to cold. COLD1^jap^ is able to interact with Gα subunit, thus activating Ca^2+^ channel for sensing low temperature and accelerating G-protein GTPase activity (Ma et al., 2015). *d1* mutant also possesses increased capability to tolerate water deficit. *d1* mutant plants exhibited decreased sensitivity to drought stress when they were exposed to progressive soil water loss (Ferrero-Serrano and Assmann, 2016). Investigations in *Arabidopsis* showed that null mutation of *Gα* subunit-encoding gene disrupted Ca^2+^-channel activation and production of reactive oxygen species (ROS) upon induction by ABA, suggesting that loss of Gα function largely abolishes ABA signaling between ABA reception and ROS production, with a consequent impairment in Ca^2+^-channel activation (Zhang et al., 2011). These studies together indicate that Gα subunit is extensively involved in plant morphological development and tolerance to different types of abiotic stresses, such as salinity, cold, drought, and ABA; however, the reason why loss of Gα function leads to enhanced tolerance to these stress factors remains largely unknown. In this study, we observed that *idr1–1* mutant plants exhibited increased tolerance to osmotic stress as they did under drought stress conditions (Zu et al., 2021). To understand the molecular basis of this phenomenon, we conducted RNA sequencing and analyzed the RNA-seq data. Our transcriptomic analyses showed that wild-type IAPAR9- and *idr1-1*-associated transcriptomic changes were stress stage-dependent (**Figure 3**). Under early-stage osmotic stress, the numbers of upregulated and downregulated DEGs in *idr1–1* mutant seedlings relative to untreated wild-type IAPAR9 seedlings (E-*idr1-1*-OS vs E-IAPAR9-CK) were 2881 and 2191, respectively; under middle-stage osmotic stress, the number of upregulated and downregulated DEGs in *idr1–1* mutant seedling (M-*idr1-1*-OS vs M-IAPAR9-CK) were 2824 and 2153, respectively (**Figures 3A, B**). This suggests that the total numbers of DEGs from both the stages appear not to alter significantly. Despite this, the DEGs (upregulated and downregulated) derived from early-stage osmotic stress (see E-*idr1-1*-OS vs E-IAPAR9-CK) were enriched mainly in the GO terms related to responses to salt, heat and cold stresses, response to ROS, ROS scavenging, stomatal movement, phytohormone biosynthesis, protein phosphorylation, etc. (**Figure 3E**); by comparison, the DEGs (upregulated and downregulated) from middle-stage osmotic stress (see M-*idr1-1*-OS vs M-IAPAR9-CK) were enriched mainly in the GO terms related to salt, heat and drought stresses, responses and regulation of phytohormones (ABA and JA), response to ROS, oxidation-reduction processes, photosynthetic system-associated components (including thylakoid, assembly and repair of photosystem I and II, photosystem II oxygen evolving complex) (**Figure 3F**). So, it appears that GO terms change from rapid responses to osmotic stress (including responses to phytohormones, response and scavenging of ROS, and protein phosphorylation) to reestablishment of oxidation-reduction balance and maintenance of photosynthetic systems (**Supplementary Figure 3**). In addition, analyses of overlapping DEGs co-regulated by *idr1–1* mutation and osmotic stress further revealed that the GO terms associated with maintenance as well as repair of photosynthetic systems and synthesis as well as activity of secondary metabolites were overrepresented, again suggesting that both processes play important roles in the responses to osmotic stress, especially for osmotic stress occurring at the middle stage (**Figures 4C, D**). Therefore, *idr1–1* mutation seems to alter photosynthetic systems and secondary metabolisms to cope with prolonged osmotic stress, which may be beneficial to the resilience of *idr1–1* mutant seedlings to osmotic stress. It is worth noting that the transcriptomic changes observed in *idr1–1* mutant seedlings may be the direct and indirect consequences of the *idr1–1* mutation. IDR1/RGA1/Gα was reported to be involved in multiple signaling pathways, including phytohormone signaling, stress-response signaling, and developmental signaling. Therefore, mutation of *IDR1* may lead to expression changes of a broad range of genes through distinct signaling pathways. As a result, the DEGs identified in *idr1–1* mutant seedlings seem to be a mixture of genes that are involved in responsiveness to osmotic stress or not. In order to overcome this shortcoming, we identified the DEGs that are co-regulated by *idr1–1* mutation and osmotic stress to exclude the DEGs that are not directly associated with responsiveness to osmotic stress to the greatest extent (**Figure 4**).

At the early and middle stages of osmotic stress, there were a total of 44 and 325 overlapping DEGs (including upregulated and downregulated ones) detected, respectively, which were co-regulated by *idr1–1* mutation and osmotic stress (**Figures 4A, B**). Of these DEGs, 24 were selected for examination of their expression levels, and heatmaps of these genes revealed that most of them showed significantly altered expression levels in *idr1–1* mutant seedlings than in IAPAR9 seedlings (**Figure 5A**). Remarkably, 5 of these DEGs, including *OsFLU1*, *OsHHO3, OsRLIN1, NADPH HC*, and *OsS40-14*, were found to interact with IDR1 as detected by Y2H and LCI assays (**Figure 6**). OsFLU1 and NADPH HC were reported to be involved in chlorophyll biosynthesis and redox regulation (Li et al., 2019). As a rice homolog of *Arabidopsis* FLU, OsFLU1 was shown to be a negative regulator of protochlorophyllide synthesis that prevents photooxidative damage during dark-to-light transitions (Li et al., 2019); the interaction between IDR1 and OsFLU1/NADPH HC may influence chloroplastic redox balance by modulating chlorophyll biosynthetic rate under osmotic stress conditions. OsHHO3, a NIGT1/HHO family transcription factor, participates in ABA- and light-mediated regulation of stomatal aperture (Li et al., 2022). Given that G protein-mediated signaling and ABA signaling pathways both influence stomatal behaviors, such an interaction between IDR1 and OsHHO3 may hint at a regulatory crosstalk between G-protein signaling and ABA- or light-mediated stomatal actions. OsRLIN1 has been documented to mediate light-dependent lesion formation caused by excessive ROS accumulation (Sun et al., 2011). Thus, the interaction of IDR1 with OsRLIN1 suggests a potential mechanistic link between G-protein signaling and tetrapyrrole-mediated oxidative regulation, implying that IDR1 may help modulate chloroplast-derived ROS homeostasis under sustained osmotic stress conditions. OsS40–14 was known as a nuclear transcriptional activator involved in regulating leaf senescence and carbon allocation (Habiba et al., 2021); therefore, the interaction of IDR1 with OsS40–14 may suppress premature senescence and maintain photosynthetic capacities of leaves. Because Gα is one of the components of G-protein signaling, it appears that G-protein signaling performs important functions in regulating chloroplast functioning and ROS homeostasis. As chloroplasts are a major source of ROS, the roles of G-protein signaling in chloroplast functioning may serve as a modulator to fine-tune ROS levels, thus preventing excessive ROS accumulation. Thus, the interactions between IDR1 and the above-mentioned 5 proteins may be related to restriction of chloroplast-derived ROS overaccumulation, maintenance or regulation of chloroplastic redox balance, stomatal actions, and premature senescence under osmotic stress conditions, thereby weakening physiological damage to *idr1–1* mutant seedlings.

## Conclusions

In conclusion, PEG-induced osmotic stress upregulated/downregulated the expression of a large collection of DEGs, including 2881 upregulated and 2191 downregulated DEGs in *idr1–1* mutant seedlings undergoing early-stage osmotic stress relative to wild-type IAPAR9 seedlings without osmotic stress; the prolonged treatment also gave rise to a substantial group of upregulated or downregulated DEGs, including 2824 upregulated and 2153 downregulated DEGs in *idr1–1* mutant seedlings undergoing middle-stage osmotic stress relative to wild-type IAPAR9 seedlings without osmotic stress. In the early stage of osmotic stress, the GO term analyses of DEGs revealed a significant enrichment of genes mainly associated with response to ROS, response to salt, heat and cold stress, phytohormone biosynthesis and signaling, stomatal movement, and post-translational protein modifications; by contrast, in the middle stage of osmotic stress, the GO term analyses of DEGs showed a significant enrichment of genes mainly related to response to ROS, response to salt and drought stress, phytohormone biosynthesis and signaling, photosystem complex assembly and repair, chlorophyll catabolic process, photorespiration, oxidoreductase activity, and thylakoid. Further analysis demonstrated that DEGs derived from the treatment by early-stage osmotic stress, which were co-regulated by osmotic stress and *idr1–1* mutation, were enriched in GO terms mainly associated with photosystem maintenance and repair, chloroplast functioning, response to phytohormones, and synthesis of secondary metabolites; by comparison, the co-regulated DEGs derived from the treatment by middle-stage osmotic stress, were enriched in GO terms mainly related to the structural integrity of chloroplasts and the operation of photosystems. Moreover, IDR1 was also able to interact with 5 proteins, i.e. OsFLU1, OsHHO3, OsRLIN1, NADPH HC and OsS40-14, whose coding genes were all co-regulated by osmotic stress and *idr1–1* mutation in the early and middle stages of osmotic stress. Altogether, our results revealed that *idr1–1* mutation caused changes in expression levels of a large number of genes, and that shifts in transcriptomic profiles occurred to some extent from early-stage osmotic stress to middle-stage osmotic stress, thereby allowing *idr1–1* mutant plants to adapt themselves to aggravated osmotic stress.

## Data Availability

The datasets presented in this study can be found in online repositories. The names of the repository/repositories and accession number(s) can be found in the article/**Supplementary Material**.

## References

[B1] Ashikari M. Wu J. Yano M. Sasaki T. Yoshimura A. (1999). Rice gibberellin-insensitive dwarf mutant gene Dwarf 1 encodes the alpha-subunit of GTP-binding protein. Proc. Natl. Acad. Sci. U.S.A. 96, 10284–10289. doi: 10.1073/pnas.96.18.10284. PMID: 10468600 PMC17880

[B2] Cao J. J. Liu C. X. Shao S. J. Zhou J. (2020). Molecular mechanisms of autophagy regulation in plants and their applications in agriculture. Front. Plant Sci. 11, 618944. doi: 10.3389/fpls.2020.618944. PMID: 33664753 PMC7921839

[B3] Chakravorty D. Trusov Y. Zhang W. Acharya B. R. Sheahan M. B. McCurdy D. W. . (2011). An atypical heterotrimeric G-protein γ-subunit is involved in guard cell K^+^-channel regulation and morphological development in Arabidopsis thaliana. Plant J. 67, 840–851. doi: 10.1111/j.1365-313X.2011.04638.x. PMID: 21575088

[B4] Chen K. Li G.-J. Bressan R. A. Song C. P. Zhu J. K. Zhao Y. (2020). Abscisic acid dynamics, signaling, and functions in plants. J. Integr. Plant Biol. 62, 25–54. doi: 10.1111/jipb.12899. PMID: 31850654

[B5] Chen H. Zou Y. Shang Y. Lin H. Wang Y. Cai R. . (2008). Firefly luciferase complementation imaging assay for protein-protein interactions in plants. Plant Physiol. 146, 323–324. doi: 10.1104/pp.107.111740. PMID: 18065554 PMC2245818

[B6] Fan L. M. Zhang W. Chen J. G. Taylor J. P. Jones A. M. Assmann S. M. (2008). Abscisic acid regulation of guard-cell K^+^ and anion channels in Gbeta- and RGS-deficient Arabidopsis lines. Proc. Natl. Acad. Sci. U.S.A. 105, 8476–8481. doi: 10.1073/pnas.0800980105. PMID: 18541915 PMC2448861

[B7] Ferrero-Serrano Á. Assmann S. M. (2016). The α-subunit of the rice heterotrimeric G protein, RGA1, regulates drought tolerance during the vegetative phase in the dwarf rice mutant d1. J. Exp. Bot. 67, 3433–3443. doi: 10.1093/jxb/erw183. PMID: 27194741 PMC4892740

[B8] Gong N. Han Y. Wang Z. Wang Y. Du Y. Chen Z. . (2025). Research progress on genes and regulatory mechanisms of drought resistance in rice. Plant Sci. 360, 112687. doi: 10.1016/j.plantsci.2025.112687. PMID: 40738192

[B9] Habiba Xu J. Gad A. G. Luo Y. Fan C. Uddin J. B. G. . (2021). Five OsS40 family members are identified as senescence-related genes in rice by reverse genetics approach. Front. Plant Sci. 12, 701529. doi: 10.3389/fpls.2021.701529. PMID: 34539694 PMC8446524

[B10] He B. Cui X. Wang H. Chen A. (2014). Drought: The most important physical stress of terrestrial ecosystems. Acta Ecol. Sin. 34, 179–183. doi: 10.1016/j.chnaes.2014.05.004. PMID: 38826717

[B11] Hu X. Qian Q. Xu T. Zhang Y. Dong G. Gao T. . (2013). The U-Box E3 ubiquitin ligase TUD1 functions with a heterotrimeric G α subunit to regulate brassinosteroid-mediated growth in rice. PloS Genet. 9, e1003391. doi: 10.1371/journal.pgen.1003391. PMID: 23526892 PMC3597501

[B12] Huo Q. Song R. Ma Z. (2024). Recent advances in exploring transcriptional regulatory landscape of crops. Front. Plant Sci. 15, 1421503. doi: 10.3389/fpls.2024.1421503. PMID: 38903438 PMC11188431

[B13] Iqbal O. Yang X. Wang Z. Li D. Wen J. Ding J. . (2025). Comparative transcriptome and genome analysis between susceptible Zhefang rice variety Diantun 502 and its resistance variety Diantun 506 upon Magnaporthe oryzae infection. BMC Plant Biol. 25, 341. doi: 10.1186/s12870-025-06357-5. PMID: 40091040 PMC11912658

[B14] Islam F. Khan M. S. S. Ahmed S. Ikram A. U. Hannan F. Jan M. . (2024). Transcriptomic reprogramming of rice cultivars in response to herbicide, salt and their combined stresses. Plant Stress 12, 100504. doi: 10.1016/j.stress.2024.100504. PMID: 38826717

[B15] Jiang Z. van Zanten M. Sasidharan R. (2025). Mechanisms of plant acclimation to multiple abiotic stresses. Commun. Biol. 8, 655. doi: 10.1038/s42003-025-08077-w. PMID: 40269242 PMC12019247

[B16] Kesawat M. S. Satheesh N. Kherawat B. S. Kumar A. Kim H. U. Chung S. M. . (2023). Regulation of reactive oxygen species during salt stress in plants and their crosstalk with other signaling molecules-current perspectives and future directions. Plants (Basel) 12, 864. doi: 10.3390/plants12040864. PMID: 36840211 PMC9964777

[B17] Khan M. I. R. Palakolanu S. R. Chopra P. Rajurkar A. B. Gupta R. Iqbal N. . (2021). Improving drought tolerance in rice: ensuring food security through multi-dimensional approaches. Physiol. Plant 172, 645–668. doi: 10.1111/ppl.13223. PMID: 33006143

[B18] Kim J. S. Kidokoro S. Yamaguchi-Shinozaki K. Shinozaki K. (2024). Regulatory networks in plant responses to drought and cold stress. Plant Physiol. 195, 170–189. doi: 10.1093/plphys/kiae105. PMID: 38514098 PMC11060690

[B19] Kumar K. N. R. Babu T. R. Hamsa K. R. Shafiwu A. B. Mahama I. (2025). Exploring the effects of climate change on rice yields in Andhra Pradesh, India. Agric. Rural Stud. 3, 1–21. doi: 10.59978/ar03010004

[B20] Li M. Kim C. (2022). Chloroplast ROS and stress signaling. Plant Commun. 3, 100264. doi: 10.1016/j.xplc.2021.100264. PMID: 35059631 PMC8760138

[B21] Li Z. Mo W. Jia L. Xu Y.-C. Tang W. Yang W. . (2019). Rice FLUORESCENT1 is involved in the regulation of chlorophyll. Plant Cell Physiol. 60, 2307–2318. doi: 10.1093/pcp/pcz129. PMID: 31290959

[B22] Li Q. Zhou L. Chen Y. Xiao N. Zhang D. Zhang M. . (2022). Phytochrome interacting factor regulates stomatal aperture by coordinating red light and abscisic acid. Plant Cell 34, 4293–4312. doi: 10.1093/plcell/koac244. PMID: 35929789 PMC9614506

[B23] Liu X. Quan W. Bartels D. (2022). Stress memory responses and seed priming correlate with drought tolerance in plants: an overview. Planta 255, 45. doi: 10.1007/s00425-022-03828-z. PMID: 35066685 PMC8784359

[B24] Livak K. J. Schmittgen T. D. (2001). Analysis of relative gene expression data using real-time quantitative PCR and the 2^-ΔΔCT^ Method. Methods 25, 402–408. doi: 10.1006/meth.2001.1262. PMID: 11846609

[B25] Ma Y. Dai X. Xu Y. Luo W. Zheng X. Zeng D. . (2015). COLD1 confers chilling tolerance in rice. Cell. 160, 1209–1221. doi: 10.1016/j.cell.2015.01.046. PMID: 25728666

[B26] Martarello D. C. I. Grizza L. H. E. Foletto-Felipe M. P. Mendonça A. S. Constantin R. P. Ferro A. P. . (2024). S-Benzyl-L-cysteine inhibits growth and photosynthesis, and triggers oxidative stress in Ipomoea grandifolia. Agronomy 14, 1633. doi: 10.3390/agronomy14081633. PMID: 30654563

[B27] Nilson S. E. Assmann S. M. (2010). Heterotrimeric G proteins regulate reproductive trait plasticity in response to water availability. New Phytol. 185, 734–746. doi: 10.1111/j.1469-8137.2009.03120.x. PMID: 20028470

[B28] Ning X. Sun Y. Wang C. Zhang W. Sun M. Hu H. . (2018). A rice CPYC-type glutaredoxin OsGRX20 in protection against bacterial blight, methyl viologen and salt stresses. Front. Plant Sci. 9, 111. doi: 10.3389/fpls.2018.00111. PMID: 29479359 PMC5811478

[B29] Sato H. Mizoi J. Shinozaki K. Yamaguchi-Shinozaki K. (2024). Complex plant responses to drought and heat stress under climate change. Plant J. 117, 1873–1892. doi: 10.1111/tpj.16612. PMID: 38168757

[B30] Schwenkert S. Fernie A. R. Geigenberger P. Leister D. Möhlmann T. Naranjo B. . (2022). Chloroplasts are key players to cope with light and temperature stress. Trends Plant Sci. 27, 577–587. doi: 10.1016/j.tplants.2021.12.004. PMID: 35012879

[B31] Song F. Yang Q. Huang J. Guo Z. Li Y. Deng W. (2026). Plant drought stress: physiological, biochemical and molecular mechanisms. Plant Stress 19, 101153. doi: 10.1016/j.stress.2025.101153. PMID: 38826717

[B32] Steffens B. Sauter M. (2009). Heterotrimeric G protein signaling is required for epidermal cell death in rice. Plant Physiol. 151, 732–740. doi: 10.1104/pp.109.142133. PMID: 19656904 PMC2754641

[B33] Sun C. Liu L. Tang J. Lin A. Zhang F. Fang J. . (2011). RLIN1, encoding a putative coproporphyrinogen III oxidase, is involved in lesion initiation in rice. J. Genet. Genomics 38, 29–37. doi: 10.1016/j.jcg.2010.12.001. PMID: 21338950

[B34] Tavu L. E. J. Redillas M. C. F. R. (2025). Oxidative stress in rice (Oryza sativa): mechanisms, impact, and adaptive strategies. Plants 14, 1463. doi: 10.3390/plants14101463. PMID: 40431027 PMC12114693

[B35] Ueguchi-Tanaka M. Fujisawa Y. Kobayashi M. Ashikari M. Iwasaki Y. Kitano H. . (2000). Rice dwarf mutant d1, which is defective in the α subunit of the heterotrimeric G protein, affects gibberellin signal transduction. Proc. Natl. Acad. Sci. U.S.A. 97, 11638–11643. doi: 10.1073/pnas.97.21.11638. PMID: 11027362 PMC17253

[B36] Urano D. Colaneri A. Jones A. M. (2014). Gα modulates salt-induced cellular senescence and cell division in rice and maize. J. Exp. Bot. 65, 6553–6561. doi: 10.1093/jxb/eru372. PMID: 25227951 PMC4246186

[B37] Wang X. Q. Ullah H. Jones A. M. Assmann S. M. (2001). G protein regulation of ion channels and abscisic acid signaling in Arabidopsis guard cells. Science 292, 2070–2072. doi: 10.1126/science.1059046. PMID: 11408655

[B38] Wang Q. Yue J. Yan J. (2023). Research progress on maintaining chloroplast homeostasis under stress conditions: a review. Acta Biochim. Biophys. Sin. (Shanghai) 55, 173–182. doi: 10.3724/abbs.2023022. PMID: 36840466 PMC10157539

[B39] Zait Y. Ferrero-Serrano Á. Assmann S. M. (2021). The α subunit of the heterotrimeric G protein regulates mesophyll CO_2_ conductance and drought tolerance in rice. New Phytol. 232, 2324–2338. doi: 10.1111/nph.17730. PMID: 34515342 PMC9293471

[B40] Zargar S. M. Mir R. A. Ebinezer L. B. Masi A. Hami A. Manzoor M. . (2021). Physiological and multi-omics approaches for explaining drought stress tolerance and supporting sustainable production of rice. Front. Plant Sci. 12, 803603. doi: 10.3389/fpls.2021.803603. PMID: 35154193 PMC8829427

[B41] Zhang L. Hu G. Cheng Y. Huang J. (2008). Heterotrimeric G protein alpha and beta subunits antagonistically modulate stomatal density in Arabidopsis thaliana. Dev. Biol. 324, 68–75. doi: 10.1016/j.ydbio.2008.09.008. PMID: 18834874

[B42] Zhang W. Jeon B. W. Assmann S. M. (2011). Heterotrimeric G-protein regulation of ROS signalling and calcium currents in Arabidopsis guard cells. J. Exp. Bot. 62, 2371–2379. doi: 10.1093/jxb/erq424. PMID: 21262908

[B43] Zheng X. Jehanzeb M. Habiba Zhang Y. Li L. Miao Y. (2019). Characterization of S40-like proteins and their roles in response to environmental cues and leaf senescence in rice. BMC Plant Biol. 19, 174. doi: 10.1186/s12870-019-1767-1. PMID: 31046677 PMC6498481

[B44] Zheng Z. Lv M. Wang Z. Du J. Xue K. Cui X. . (2026). Phenological stage-dependent hierarchical responses mediate extreme drought impacts on carbon fluxes of a semiarid grassland. New Phytol. 249, 1204–1218. doi: 10.1111/nph.70739. PMID: 41246909

[B45] Zhu C. Q. Ye Y. X. Qiu T. Huang Y. F. Ying J. F. Shen Z. C. (2024). Drought-tolerant rice at molecular breeding eras: an emerging reality. Rice Sci. 31, 179–189. doi: 10.1016/j.rsci.2023.11.005. PMID: 38826717

[B46] Zu X. Lu Y. Wang Q. La Y. Hong X. Tan F. . (2021). Increased Drought Resistance 1 mutation increases drought tolerance of upland rice by altering physiological and morphological traits and limiting ROS levels. Plant Cell Physiol. 62, 1168–1184. doi: 10.1093/pcp/pcab053. PMID: 33836080

