# Supplementary material for: Mechanisms behind idr1–1 mutation conferring osmotic-stress tolerance to rice seedlings as revealed by stage-based transcriptomes
Source: Front Plant Sci. 2026 May 13;17:1815995. doi: 10.3389/fpls.2026.1815995 (PMC13212348; doi:10.3389/fpls.2026.1815995)
Supplement: Supplementary file 1 [file DataSheet1.docx]

**Supplementary Figures and legends**

**
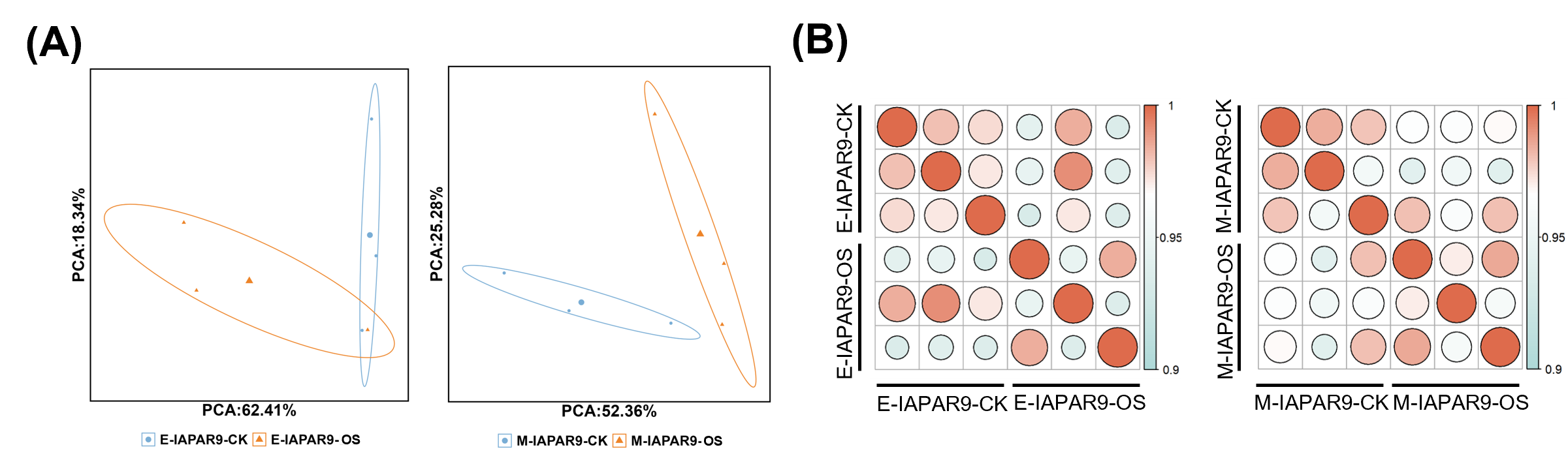
**

**Figure S1. Examination of quality of mRNA-seq data derived from IAPAR9 and *idr1-1* mutant seedlings experiencing early or middle stage of osmotic stress.**

**(A)** PCA of the mRNA-seq data derived from wild-type IAPAR9 seedlings under control (nontreatment) or osmotic-stress treatment conditions.

**(B)** Pair-wise Pearson’s correlation analyses of mRNA-seq data sets as indicated. Analyses were performed using the CPM-normalized expression matrix after filtering genes with CPM < 10.


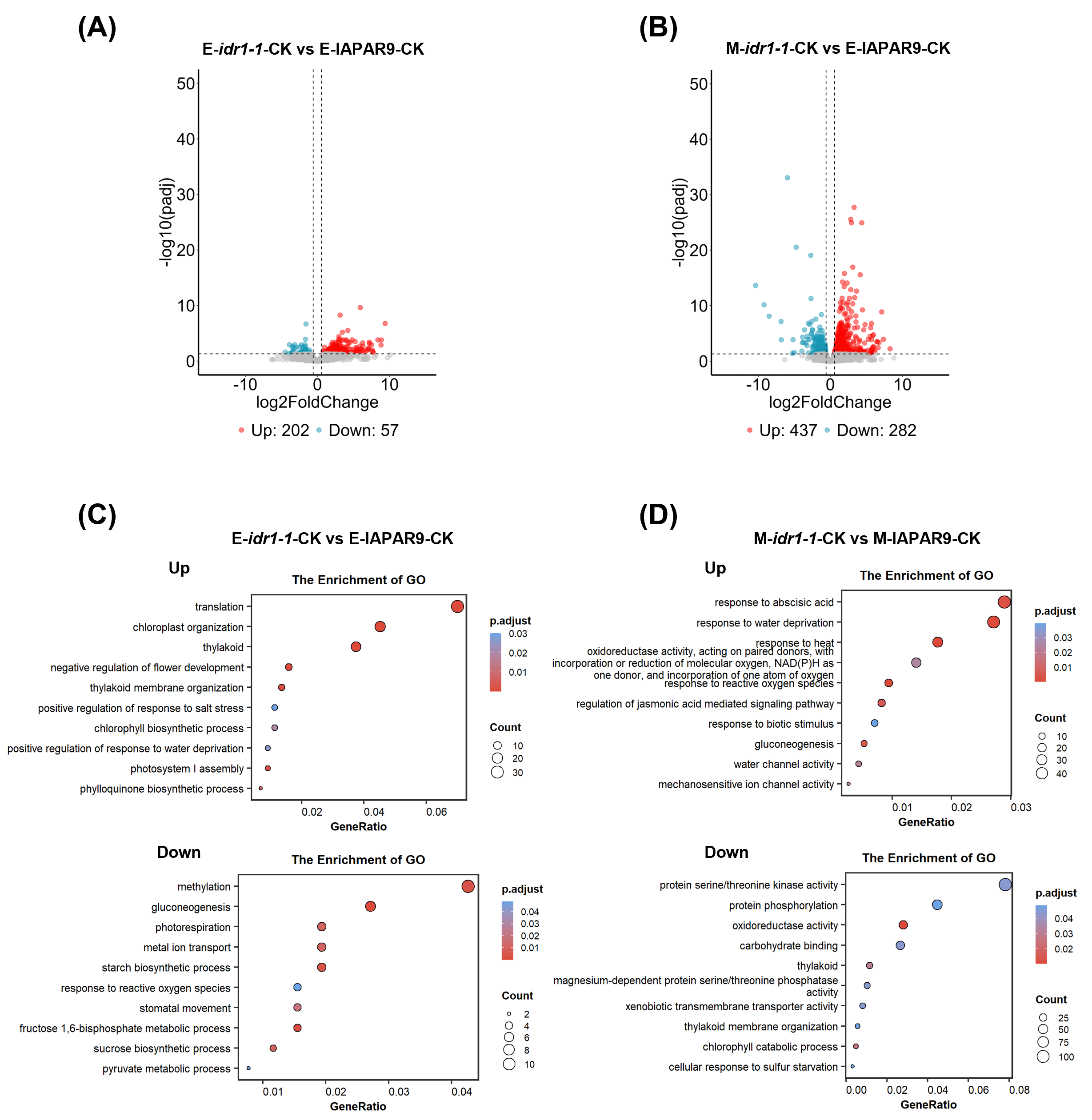


**Figure S2. Identification of upregulated and downregulated DEGs under control conditions and GO term analyses of the DEGs.**

**(A-B)** Volcano plots showing DEGs identified from *idr1-1* mutant seedlings versus wild-type IAPAR9 seedlings under the early **(A)** or middle **(B)** stage of nontreatment (control) conditions. Red and blue dots indicate significantly upregulated and downregulated DEGs, respectively, while gray dots indicate non-significant DEGs. Dashed vertical and horizontal lines denote thresholds to judge statistical significance (false discovery rate (FDR) < 0.05 and |Fold Change| ≥ 1.5). The numbers of up- or downregulated DEGs are given below each plot.

**(C-D)** GO enrichment analyses of DEGs coming from the comparison group E-*idr1-1*-CK vs E-IAPAR9-CK in **(A) (C)**, and from comparison group M-*idr1-1*-CK vs E-IAPAR9-CK in **(B) (D)**. The upregulated and downregulated DEGs were separately used for GO enrichment analyses, and thus denoted as “Up” and “Down”, respectively. Dot size represents the number of DEGs in each GO term, and color indicates the adjusted *P* value for enrichment.


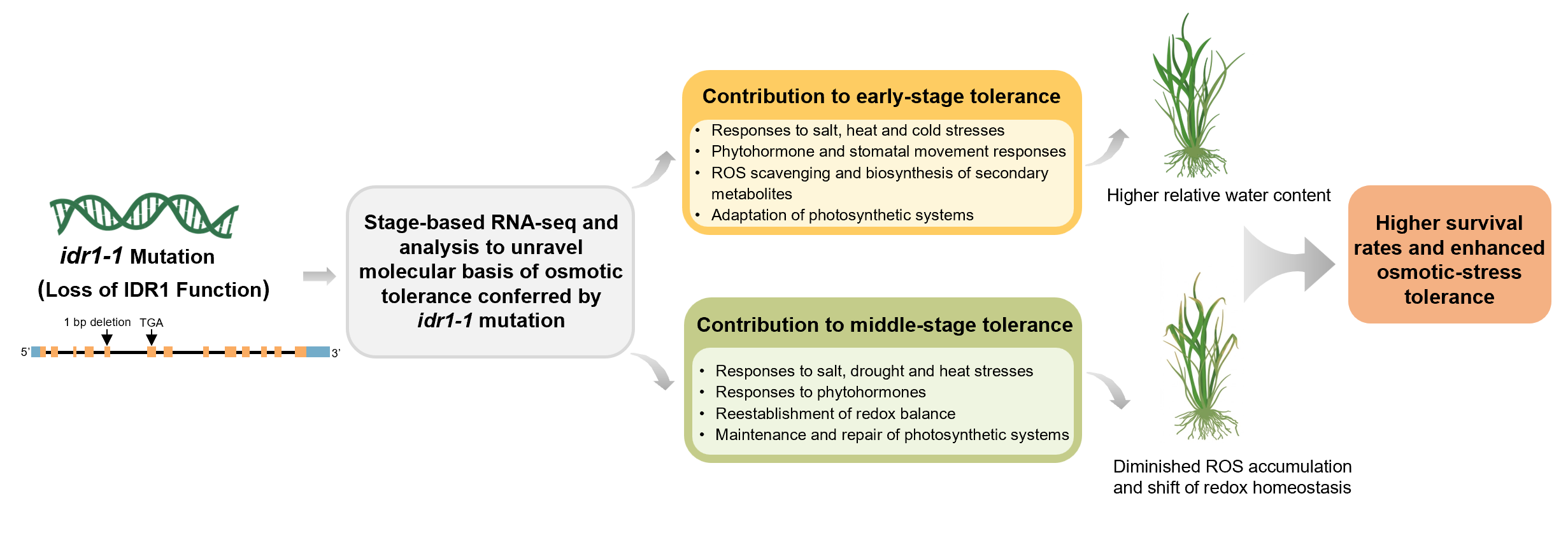


**Figure S3. A diagram of how *idr1-1* mutation confers enhanced tolerance to osmotic stress in rice under early and middle stages of osmotic stresses.**

Loss of IDR1 function in *idr1-1* mutant is rooted in a 1-bp deletion on the 5th coding exon, which causes a frameshift mutation and simultaneously creates a stop codon on the 6th coding exon. To learn about how *idr1-1* mutation increases tolerance to osmotic stress in rice, stage-based RNA sequencing and analyses with *idr1-1* mutant and wild-type IAPAR9 seedlings were conducted. The analyses revealed that *idr1-1* mutation made contributions to enhanced osmotic-stress tolerance in different ways: in the early stage of osmotic stress, the enhancement of tolerance was mainly associated with 4 physiological processes, including responses to salt, heat and cold stresses, phytohormone and stomatal movement responses, ROS scavenging and biosynthesis of secondary metabolites, and adaption of photosynthetic systems; in the middle stage of osmotic stress, the enhancement of tolerance was chiefly associated with 4 physiological processes too: responses to salt, drought and heat stresses, responses to phytohormones, reestablishment of redox balance, and maintenance and repair of photosynthetic systems. The responses to early-stage osmotic stress allowed seedlings to retain relatively higher water contents, and those to middle-stage osmotic stress allowed lower ROS accumulation and shifts of redox homeostasis. All of these alterations occurring in *idr1-1* mutant seedlings improved survival rates of the *idr1-1* mutant seedlings, thereby making them survive prolonged osmotic stress.
